# Documenting lancet core surgical indicator on volumes: a scoping review of data collection methods

**DOI:** 10.1136/bmjopen-2026-118534

**Published:** 2026-11-02

**Authors:** Priti Patil, Alex van Duinen, Juul Bakker, Priyansh Nathani, Yuki J Ng, Monali Mohan, Pranav Bhushan, Sidharth Misra, Karel Cornelis Lindenbergh, Håkon A Bolkan, Nobhojit Roy, Anita Gadgil

**Affiliations:** 1 Department of Statistics, BARC Hospital, Mumbai, India; 2 Department of Public Health and Nursing, Norwegian University of Science and Technology (NTNU), Trondheim, Trøndelag, Norway; 3 CapaCare, Trondheim, Norway; 4 Department of Surgery, St. Olavs Hospital HF, Trondheim University Hospital, Trondheim, Norway; 5 ELWA Hospital, Monrovia, Liberia; 6 WHO Collaborating Centre for Emergency, Critical and Operative Care, The George Institute for Global Health India, New Delhi, India; 7 Department of General Surgery, University Hospital Bristol, Bristol, UK; 8 Indraprastha Apollo Hospitals, New Delhi, India; 9 Department of Public Health, Institute of Global Public Health, University of Manitoba, Winnipeg, Manitoba, Canada; 10 Armed Forces Medical College, Pune, India; 11 Department of Surgery, The University of Texas Southwestern Medical Center, Dallas, Texas, USA; 12 Doctors of the World the Netherlands, Amsterdam, The Netherlands; 13 Department of Global Public Health, Karolinska Institutet, Stockholm, Sweden; 14 University of Global Health Equity, Butaro, Rwanda; 15 WHO Collaborating Centre for Emergency, Critical and Operative Care, The George Institute for Global Health, New Delhi, India

**Keywords:** SURGERY, PUBLIC HEALTH, Health policy, Electronic Health Records, Data Accuracy

## Abstract

**Abstract:**

**Objectives:**

Surgical volume serves as a key indicator of healthcare system capacity and performance. The accuracy and reliability of reporting surgical volumes depend on the methodology used to collect and analyse data. In this scoping review, we aim to map the methodologies used to collect data on surgical volume and assess their strengths and limitations.

**Design:**

A scoping review reported in accordance with the Preferred Reporting Items for Systematic Reviews and Meta-Analyses for Scoping Review (PRISMA-ScR) guidelines.

**Data source:**

Peer-reviewed and grey literature were searched in PubMed, Scopus, Web of Science, Cochrane Library, CINAHL, EMBASE, Google Scholar, Google and other relevant grey literature sources.

**Eligibility criteria for selecting studies:**

We included original studies and grey literature published between January 2014 and April 2026 describing the methodologies used to collect data for estimating surgical volume, irrespective of the healthcare setting or geographical region.

**Data extraction and synthesis:**

Two reviewers independently screened studies in two stages (title/abstract and full-text), with disagreements resolved by a third reviewer. Data were extracted using a standardised Excel-based form and synthesised using descriptive methods.

**Result:**

Of 12 743 records identified, 60 original studies and 22 grey literature sources were included. Substantial methodological heterogeneity was observed in the estimation of surgical volume. Paper-based operative logbooks and retrospective hospital records were the predominant data sources, while electronic medical records and administrative databases were more common in high-income settings. Important variations were also identified in the definitions of surgery, coding practices, data validation, missing-data reporting and healthcare sector coverage.

**Conclusion:**

This review identified important methodological gaps in the estimation of surgical volume. Developing standardised and comprehensive approaches to data collection and reporting will improve the completeness, reliability and comparability of surgical volume estimates for health system planning and monitoring.

**STRENGTHS AND LIMITATIONS OF THE STUDY**

- This scoping review addresses an important evidence gap by comprehensively mapping the various methodologies used to estimate surgical volume, including data collection approaches, data sources, coding practices, validation processes and reporting systems across different healthcare settings.
- The review followed Preferred Reporting Items for Systematic Reviews and Meta-Analyses for Scoping Review (PRISMA-ScR) guidelines and used a comprehensive search strategy with a standardised study selection and data extraction process.
- The inclusion of grey literature enabled the identification of national reports, policy documents and health information system resources that are often unavailable through bibliographic databases.
- Despite an extensive search, some unpublished or non-publicly accessible reports may not have been identified, potentially resulting in the omission of relevant methodological approaches.
- The review was limited to studies published in English, which may have resulted in the omission of relevant evidence published in other languages.

## Introduction

 Globally, an estimated 5 billion people lack access to safe, timely and affordable surgical and anaesthesia care when needed.^1^ The burden is greatest in low- and middle-income countries (LMICs), where inadequate access to essential surgical services is estimated to result in approximately 4.7 million avoidable deaths each year.^2^ The Lancet Commission on Global Surgery (LCoGS) has identified six indicators for monitoring access to safe, affordable surgical and anaesthesia care. These include timely access to surgery, the number of surgical procedures performed annually per 100 000 population, specialist surgical workforce density, perioperative mortality rate and protection against impoverishing and catastrophic expenditures. These indicators provide a comprehensive framework for assessing the capacity, performance and effectiveness of surgical systems globally.^1^

The LCoGS has set a target of a surgical volume of 5000 procedures per 100 000 population by 2030 for the delivery of essential surgical and anaesthesia care.^1 3^ Surgical volume is defined as the number of procedures performed in an operating theatre (OT) under regional or general anaesthesia per 100 000 population per year in a region or country.^1 4^ It has also been adopted by the World Bank and is included in its World Development Indicators.^5 6^ Robust data on surgical volume help to identify gaps in access, assess trends and evaluate the effectiveness of health interventions. However, the accuracy and reliability of such data depend on the methodology used to collect it. Despite this, limited attention has been paid to different methodologies used to collect and estimate surgical volume data across different settings. Previous studies and reviews have primarily focused on reporting surgical volume estimates with little emphasis on the underlying data-collection approaches, validation processes, data sources and reporting systems used to generate these estimates. This variability in methodologies can lead to inconsistent and non-comparable data, hindering efforts to assess surgical capacity in different regions and populations.^7^ Despite the importance of surgical volume as a global health indicator, methodological approaches to collecting and estimating surgical volume have not been systematically synthesised across settings.

This scoping review therefore aims to address this gap by systematically examining the various methodologies used to collect data on surgical volume and assessing their strengths and limitations. The findings will be of interest to policymakers, healthcare administrators and researchers working to enhance surgical care delivery and equity worldwide.

## Methodology

We reported this scoping review in accordance with the Preferred Reporting Items for Systematic Reviews and Meta-Analyses for Scoping Review (PRISMA-ScR) guidelines.^8^

### Search strategy

A comprehensive literature search was conducted in MEDLINE (via PubMed), Scopus, Web of Science, Cochrane Library, CINAHL and EMBASE (embase.com) for articles published between January 2014 and April 2026. The search was limited to studies published from January 2014 onwards to capture methods that reflect current surgical data collection practices and health information systems. The population-concept-context strategy recommended by the Joanna Briggs Institute (JBI) was applied.^9^ This review focused on sources reporting surgical volume (Population), the methodologies used to collect and estimate surgical volume data (Concept) and healthcare settings across all geographical and health system contexts (Context). Accordingly, the search strategy was designed using terms related to surgical volume, surgical rate, surgical enumeration, surgical measurement and surgical need, with Boolean operators such as ‘AND’ and ‘OR’ (online supplemental table 1). In addition to the systematic database searches, a structured grey literature search was conducted to identify unpublished and non-peer-reviewed documents describing methodologies for collecting surgical volume data. Sources like international organisation websites (eg, WHO, World Bank), Ministries of Health, professional surgical organisations, academic repositories, Google Scholar and Google were included. Search strategies included combinations of “surgical volume”, “surgery report”, “indicator”, “metadata”, “clinical registry”, “national registry”, and “health information system”. Google searches used advance operators like ‘site:’ and ‘filetype:pdf’. The same eligibility criteria for study selection and data extraction process as for peer-reviewed studies were applied to grey literature documents.

**Table 1:** Inclusion/exclusion criteria

Inclusion criteria	Exclusion criteria
Studies were included if they described how data were collected to estimate surgical volume or related quantitative measures of surgical activity. These measures included the total number of surgical procedures performed, procedure-specific surgical volumes, population-based surgical rates, hospital operative caseloads and national or subnational estimates of surgical volume.	Studies that were related to surgical volume but did not describe the data collection methodology.
Studies published between January 2014 and April 2026.	Studies focusing on procedure-specific outcomes or disease-specific outcomes without describing methods for estimating surgical volume.
Studies conducted across multiple levels of the health system, including tertiary, district, regional and national settings.	Studies that describe surgical volume in relation to outcomes, such as postoperative morbidity, mortality, length of hospital stay, time to recovery, patient-reported quality of life, functional status, satisfaction with surgery, long-term survival, recurrence rates or the need for additional surgeries.
Studies employing qualitative, quantitative, mixed-methods and modelling approaches were eligible for inclusion. Quantitative study designs include cross-sectional, cohort, survey-based and registry-based studies.	Editorials, opinion pieces, randomised controlled trials, letters to the editor, conference abstracts and commentaries.
Studies based on both primary and secondary data sources were eligible. Primary data sources referred to directly collected data such as hospital records, operative logbooks and surveys, whereas secondary data sources included registries, administrative databases, HMIS, DHS and other existing datasets.	Studies without sufficient methodological detail describing data collection methods.
Studies conducted at multicentre settings as well as single institutions were eligible if they described methods used for the collection or estimation of surgical volume data.	
Studies published in English language only due to resource limitation related to the translation and screening of non-English literature.	

DHS, Demographic and Health Survey; HIMS, Health Management Information Systems.

### Inclusion-exclusion criteria

Studies that discussed the data collection methodologies for surgical volume at the patient, facility, regional or national level were included. The detailed inclusion/exclusion criteria are stated in table 1.

### Study selection

Study screening was conducted in pairs by PP, AvD, JB, MM, PN, KCL, SM and PB. To ensure an equitable workload, all articles were distributed evenly among four pairs of reviewers. Screening was conducted in two stages: first by title and abstract, followed by full-text screening. Within each pair, both authors independently screened the assigned studies to ensure a comprehensive and unbiased selection process. Discrepancies or uncertainties, if any, were resolved by a third independent reviewer (HAB, NR, AG, AvD and JB) assigned to each group. Two reviewers (AvD and JB) participated in both screening and conflict resolution but were assigned to resolve conflicts in groups other than their initial screening assignments.

Screening was performed using the Rayyan web-based application, developed by the Qatar Computing Research Institute (QCRI), to facilitate a streamlined and systematic review process.^9^ Screening was conducted in blind mode to reduce bias, ensuring reviewers made independent decisions without influence from other reviewers’ selections. After the two primary reviewers completed the screening process, blind mode was turned off to access the conflicting articles. At this stage, the third reviewer resolved the discrepancies. If the conflict persisted, it was addressed and resolved during weekly author group discussion meetings.

### Data extraction and synthesis

A structured process for data extraction was implemented. An Excel-based data extraction sheet was developed and piloted in two rounds to refine it and ensure that all co-authors understood the process. In the first pilot round, all authors extracted data from the same two studies. The extraction sheet was then refined and inconsistencies addressed. In the second pilot round, each author independently extracted data from two different studies to ensure consistency in the extraction process. Findings from these two pilot rounds were used to identify gaps in the extraction process, which were addressed during weekly team calls and used to refine the data extraction sheet.

The final data extraction sheet contained five parts: (i) Study characteristics included the study title, publication year, study design, country, setting and health system characteristics. (ii) Database characteristics included the name of the registry or database (if any), the type of database and the population covered, such as national or sub-national population. (iii) Data collection methods included who collected the data, the duration of the data captured, the source/mechanism of the data collection and the frequency of data collection. (iv) Data contents included the type of surgery and coding mechanism, if any. Finally, (v) challenges and limitations related to data collection, if mentioned in the study, were documented to provide insights into barriers and constraints in collecting surgical volume data.

All extracted data were synthesised descriptively and presented in tabular format in accordance with JBI recommendations for scoping review. Study characteristics and methodological features of included original studies and grey literature were summarised using frequencies and percentages as appropriate. Extracted information on study setting, data sources, facility type, data collection methods, definitions of surgery and surgical volume, coding mechanisms, data validation, management of missing data and common methodological limitations was grouped into predefined categories to facilitate comparison across studies. The common methodological limitations reflected both those reported by the original study authors and those identified by the review authors based on the methodological characteristics of the included sources. To explore differences in surgical volume data collection methods across healthcare settings, a descriptive comparison was undertaken between studies conducted in low- and lower-middle-income countries (LICs/LMICs) and those conducted in upper-middle- and high-income countries (UMICs/HICs), based on the World Bank income classification.^10^ Results were presented using summary tables to map the existing evidence and identify gaps in current methodologies.

## Results

### Study characteristics

A total of 82 documents were included in this scoping review. The initial search across all databases identified 12 743 records. After removing duplicates and following the screening process, 42 articles were included. An additional 18 articles were identified through citation searching and Google Scholar, for a total of 60 articles included for final data extraction and analysis (figure 1). A total of 42 records were identified through grey literature search, of which 22 were included in the review after assessment for relevance and eligibility criteria. These comprised technical reports, policy documents, indicator metadata, registry reports, health information system documentation and national strategic plans.

**Figure 1:**
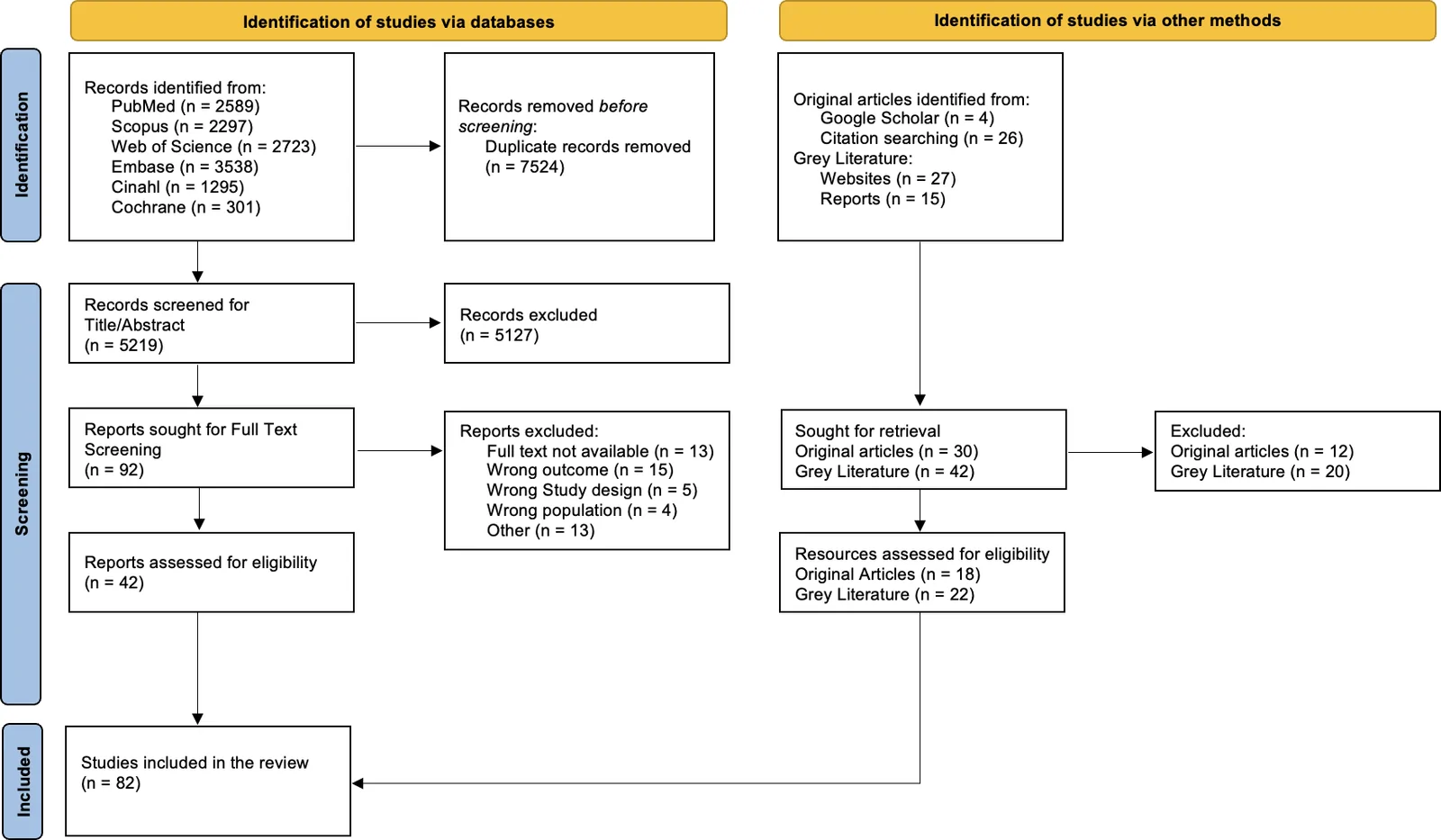
PRISMA flow diagram of the study selection process. PRISMA, Preferred Reporting Items for Systematic Reviews and Meta-Analyses.

Among the 82 included resources, 37 sources (45.1%) were conducted in Africa, followed by Asia (15.9%) and North America (14.6%) (figure 2). The majority of original studies were conducted in LMICs (68.3%), whereas grey literature included a greater proportion of high-income countries (40.9%) and multi-country sources (22.7%) reporting surgical volume data from countries across different income groups. Of all included original studies, 65% used data from both rural and urban settings. Among all included resources, 75.6% used retrospective data. Four studies (4.9%) focused on private sector institutions, while 27 (32.9%) included both public and private institutions (table 2).

**Figure 2:**
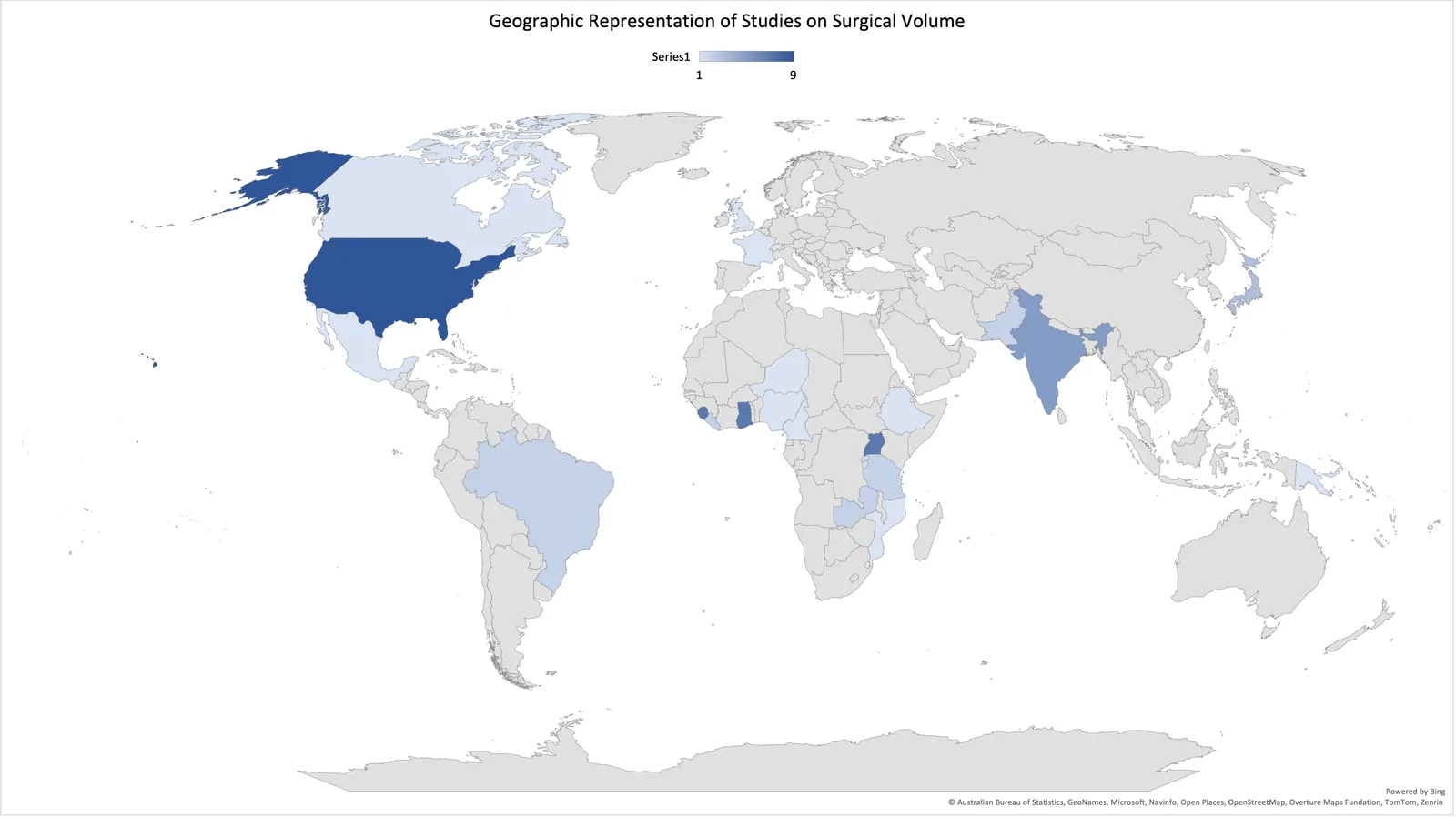
Geographic representation of studies on surgical volume.

**Table 2:** Characteristics of included original studies and grey literature

Characteristics	Original articles (n=60)	Grey literature (n=22)	Total (n=82)
Regions			
Africa	34 (56.7%)	3 (13.6%)	37 (45.1%)
Asia	7 (11.7%)	6 (27.3%)	13 (15.9%)
North America	10 (16.7%)	2 (9.1%)	12 (14.6%)
Europe	2 (3.3%)	5 (22.7%)	7 (8.5%)
South America	2 (3.3%)	1 (4.5%)	3 (3.7%)
Austrasia	1 (1.7%)	0 (0.0%)	1 (1.2%)
Multi-country	4 (6.7%)	5 (22.7%)	9 (11.0%)
Country income group*			
LIC	15 (25%)	1 (4.5%)	16 (19.5%)
LMIC	26 (43.3%)	4 (18.2%)	30 (36.6%)
UMIC	2 (3.3%)	3 (13.6%)	5 (6.1%)
HIC	14 (23.3%)	9 (40.9%)	23 (28.0%)
Multi-country	3 (5.0%)	5 (22.7%)	8 (9.8%)
Study setting			
Urban+rural	39 (65%)	NR	39 (47.6%)
Rural only	12 (20%)	NR	12 (14.6%)
Urban only	9 (15%)	NR	9 (11.0%)
Study design			
Cross-sectional	48 (80.0%)	NR	48 (58.5%)
Cohort	7 (11.7%)	NR	7 (8.5%)
Modelling	2 (3.3%)	NR	2 (2.4%)
Other	3 (5.0%)	NR	3 (3.7%)
Data collection type			
Retrospective	53 (88.3%)	9 (40.9%)	62 (75.6%)
Prospective	7 (11.7%)	10 (45.5%)	17 (20.7%)
Not reported	0 (0.0%)	3 (13.6%)	3 (3.7%)
Health system setting studied			
Public only	35 (58.3%)	6 (27.3%)	41 (50.0%)
Private only	3 (5.0%)	1 (4.5%)	4 (4.9%)
Public and private	22 (36.7%)	5 (22.7%)	27 (32.9%)
Participating facilities	0 (0.0%)	3 (13.6%)	3 (3.7%)
Not specified	0 (0.0%)	7 (31.8%)	7 (8.5%)

* Country income groups were classified according to the World Bank country income classification.

.HIC, high-income countries; LIC, low income countries; LMIC, lower-middle-income countries.; NR, not reported; UMIC, upper-middle income countries.

### Methodological characteristics of the included studies

Methodological characteristics of the 60 original studies and 22 grey literature resources are summarised in online supplemental table 2. Most original studies were based on nationally representative data (66.7%), while 30% were hospital-based. In contrast, grey literature included a broader range of data sources, incorporating nationally representative reports (31.8%), hospital-based reports (27.3%), multi-source compilations (18.2%), international datasets (13.6%) and registry-based resources (9.1%). Original studies mostly relied on trained researchers (36.7%), followed by facility personnel (33.3%) and extraction from electronic medical records (26.7%) to compile surgical volume data. Grey literature was predominantly compiled by government health authorities (45.5%). Paper-based hospital logbooks remained the most frequently used source among original studies (56.7%), whereas electronic medical records accounted for one-third of studies (33.3%). Grey literature relied more frequently on electronic medical records (40.9%), health management information systems, registry databases and secondary data compilations.

Reporting of surgical definitions was inconsistent. Most evidence sources (original studies+grey literature) reported all surgical procedures (59.8%), whereas approximately one-quarter focused on specific procedures (25.6%). Emergency, elective and essential surgery indicators were comparatively uncommon (11.0%). Of all evidence sources, 39.0% did not explicitly define surgery. Among those that did, the most frequently reported definitions were the Utstein definition of major surgery (25.6%), procedure-specific definitions (13.4%), surgery performed in an OT regardless of anaesthesia (12.2%) and study-specific definitions (9.8%). Definitions of surgical volume also varied substantially. The most common definition was the number or rate of procedures per 100 000 population (390%), consistent with the Lancet Commission on Global Surgery indicator. However, several resources reported total numbers of procedures with or without population denominators, while 13.4% did not explicitly define surgical volume. International Classification of Diseases (ICD)-based coding systems were the most frequently reported (12.2%). However, 79.3% of overall evidence sources did not specify the coding mechanism used to classify surgical procedures.

Approximately two-thirds of the included evidence (68.3%) did not describe any data validation procedures. Where reported, validation most frequently involved routine quality assurance activities such as staff training, data audits, double data entry, standardised abstraction tools and independent verification. Statistical validation methods and validation against external reference datasets were only reported occasionally (4.9%). Similarly, more than two-thirds of evidence sources (67.1%) did not describe how missing data were handled. Among studies reporting missing data handling, approaches included exclusion of incomplete records, weighting or alternative estimation techniques, multiple imputation and routine data quality monitoring. The most common methodological limitations identified across the original studies included reliance on routinely collected retrospective data (85.0%), concerns regarding generalisability (65.0%), incomplete documentation or missing records (55.0%), coding or recording errors (45.0%), restricted surgical scope (43.3%) and dependence on manual paper-based logbooks (38.3%). Among the grey literature sources, the most identified methodological limitations were restricted geographical coverage (45.5%), dependence on secondary administrative data (36.4%), lack of methodological transparency (27.3%), limited surgical scope (22.7%), heterogeneity/lack of standardisation (22.7%) and weaknesses in routine reporting systems (18.2%) (online supplemental tables 3, 4).

### Comparison between LIC/LMIC and UMIC/HIC

Distinct differences in data collection approaches were observed when examining methodological characteristics across country income groups. Among included original studies, paper-based hospital logbooks and registers were used in 31 of 41 studies (75.6%) conducted in LICs and LMICs, whereas only four studies (9.8%) used electronic medical record data. In contrast, all 16 studies conducted in UMICs and HICs relied on electronic medical records.

## Discussion

In this scoping review, we highlighted several methodological aspects and limitations in collecting data to report surgical volume. Although surgical volume has become a key indicator for monitoring access to surgical care, considerable heterogeneity was identified in the methods used to generate these estimates. Differences were observed in data sources, definitions of surgery and surgical volume, coding mechanisms, validation procedures and reporting practices across healthcare settings and country income groups by country. Although many studies reported nationally representative surgical volume estimates, the methods used to collect these data varied considerably, ranging from paper-based hospital records to electronic medical records. A systematic review focused on LMICs found that significant heterogeneity in sources, methodologies for collecting surgical volume data and inconsistent reporting have impeded comparison between countries.^11^ Collectively, this scoping review highlights important methodological gaps that may affect the completeness, comparability and interpretation of surgical volume estimates across settings. The methodological gaps identified in this review can broadly be grouped into two categories: variation in the systems used to collect surgical volume data and inconsistencies in the methods used to define, classify, validate and report these data.

One of the most prominent findings of this review was the substantial variation in data collection sources used to estimate surgical volume. Paper-based hospital logbooks remained the predominant source of surgical volume data, particularly in LMICs, whereas studies from UMICs and HICs more often relied on electronic medical records and administrative databases. Although paper records provide a practical and inexpensive way to document surgical activity, they are susceptible to incomplete documentation, transcription errors, missing information and physical deterioration. Furthermore, manual extraction of these records is labour-intensive and limits timely analysis and routine monitoring. The continued reliance on handwritten OT logbooks reflects the limited availability of digital health information systems in many resource-constrained settings. A study published by Guest *et al* in the Pacific region found that some of the countries had a system of regular reporting of surgical volume in place through a pre-existing centralised database maintained by the government, while for others this was a labour-intensive process requiring the manual collection of data from theatre logbooks in each operating facility.^12^ Compared with paper-based systems, electronic medical records facilitate real-time data retrieval, longitudinal follow-up, data validation and efficient aggregation of surgical volume information. These findings are consistent with previous reports indicating that electronic health information systems improve the timeliness, completeness and accessibility of routinely collected surgical data, while continued reliance on paper-based records poses challenges for data quality and routine monitoring.^12 13^

To achieve the LCoGS target for surgical volume by 2030 in LMICs, significant investments are needed in infrastructure, healthcare workforce training and reliable connectivity to ensure effective reporting through implementation and utilisation of electronic medical records (EMR).^14 15^

This review found an overwhelming reliance on retrospective data sources for estimating surgical volume. This review reports that 75.6% of studies from original articles and grey literature relied on retrospective data to estimate surgical volume. Most studies extracted information from existing hospital records, administrative databases or health information systems, while relatively few employed prospectively designed data collection methods. This suggests that surgical volume estimation depends largely on routine documentation practices rather than purposive surveillance specifically designed to monitor surgical activity. Consequently, the quality and completeness of the estimates are influenced by the accuracy of routine record-keeping, which may vary across facilities and settings. The limited use of prospective data collection also restricts opportunities for real-time monitoring, timely quality assurance and standardised reporting of surgical services. A prospective standardised data-collection surveillance system combined with electronic medical records could improve the completeness, reliability and comparability of surgical volume estimates over time.^13 16 17^ It will also help to monitor surgical capacity and volume continuously and evaluate temporal trends.

Another important finding of this review was inconsistency in the definitions of both surgery and surgical volume across studies. Although several studies adopted the Utstein definition of surgery, a substantial proportion either used study- or procedure-specific definitions or did not explicitly define the terms. Similar variations were observed in the definition of surgical volume, with studies reporting total number of procedures, procedure-specific volumes or hospital caseloads without consistently specifying which surgical procedures were included in the reported surgical volume estimates. The lack of standardised definitions makes it difficult to compare surgical volume estimates across studies and countries. Differences in surgical procedure definitions can lead to variations in reported surgical volume, even when similar services are being delivered. The Utstein consensus definition has been widely used in global surgery research. It defines surgery as procedures performed in an OT under any type of anaesthesia.^18^ While this definition facilitates standardisation, it may not capture the full spectrum of surgical care delivered outside conventional OTs, including bedside procedures, emergency department interventions, and other procedures performed in non-OT settings, particularly where a substantial proportion of procedures are performed outside the OT. Consequently, using this definition could lead to an underestimation of the overall surgical workload and may limit the ability of surgical volume indicators to fully reflect the burden of surgical care. More inclusive definitions of ‘surgery’ and ‘surgical volume’ are needed to address this. Standardised definitions should allow comparisons across settings and capture all clinically important surgical procedures, including those performed outside OTs.

Most studies did not specify whether standardised coding systems were used to classify surgical procedures, making it difficult to determine exactly which procedures were included in the reported surgical volume. Among studies that described coding practices, different classification systems were used, including national and international classifications as well as institution-specific coding schemes. This lack of consistent reporting of coding mechanisms reduced the comparability and reproducibility of surgical volume estimates across studies and healthcare settings. Standardised and transparent reporting of coding methods would improve the consistency and interpretation of surgical volume data.^17 18^

This review also identified another methodological gap: the limited reporting of data validation and missing-data management. The majority of studies (68.3%) did not describe whether surgical volume data were validated against other data sources. Similarly, information on the extent of missing data and the methods used to handle incomplete records was rarely reported. Only 32.9% of studies in this review reported some form of missing-data handling technique. Transparent reporting of these processes is fundamental to assessing data quality, reproducibility, and potential sources of bias. Without such information, it is difficult to ascertain whether differences in reported surgical volume reflect true variation in surgical activity or inconsistencies arising from data quality and reporting practices. This may reduce confidence in comparisons across healthcare settings and over time.^17 19 20^

A significant number of studies estimated surgical volume using data from public healthcare facilities alone, with little or no contribution from private healthcare facilities. As a result, the reported surgical volume may not fully represent the surgical services delivered within a country. In many regions, private hospitals contribute substantially to the total surgical volume, particularly for elective and specialised procedures. The exclusion of private settings results in incomplete datasets, limiting the ability to make informed comparisons and develop comprehensive health system assessments.

Similar concerns have been highlighted by Zadey *et al* in their correspondence, noting that a major limitation of estimating surgical volume using national Health Management Information Systems (HMIS) is that they often inadequately capture surgical data from private healthcare facilities. Furthermore, incomplete and poor-quality data within HMIS undermine its reliability for effective surgical planning and monitoring.^21^ Comprehensive estimation of national surgical volume requires reporting systems that capture surgical activity across all sectors providing surgical care, rather than relying solely on public-sector facilities.

This review suggests that, to improve surgical volume estimation and reporting, both data collection systems and reporting practices need to be strengthened. Priority areas include implementing electronic health information systems, promoting prospective routine surveillance of surgical activity, adopting standardised definitions and coding systems, improving reporting of validation procedures and the management of missing data, and ensuring that national estimates adequately capture surgical activity across both public and private healthcare sectors.

In addition to strengthening facility-based reporting systems, alternative approaches for estimating surgical indicators should also be explored. Studies by Juran *et al* and Zadey *et al* have highlighted the potential of incorporating surgical indicator questions into nationally representative surveys such as the Demographic and Health Survey (DHS).^22^ Given its standardised and replicable design, the DHS offers a robust platform for systematically capturing questions related to surgical indicators in a large-scale survey that generates nationally representative data. This approach can help collect consistent and comparable surgical data, which is important for improving and strengthening surgical aspects of healthcare systems.^23 24^ Apart from household surveys, many countries maintain Health Information Management Systems (HIMS) that routinely report surgical volume to national health authorities.^21 25–27^ These systems play a crucial role in monitoring the delivery of surgical services. However, their usefulness depends on the completeness, quality and representativeness of the underlying data, including adequate coverage of private healthcare facilities.

The findings of this review align with global efforts to strengthen surgical systems, including the Lancet Commission on Global Surgery and the WHO Resolution WHA68.15, which emphasise the importance of reliable surgical information systems for monitoring access to safe, timely and affordable surgical care. Standardised approaches to measuring surgical volume are essential for tracking progress towards national surgical planning and global surgery targets. Future research should evaluate the completeness, reliability and feasibility of different data collection systems across diverse healthcare settings and develop consensus-based methodological standards for estimating and reporting surgical volume. Such efforts would improve the comparability of surgical volume estimates and strengthen their utility for monitoring surgical system performance and informing health policy.

### Strengths and limitations

A major strength of this review was the inclusion of both peer-reviewed studies and grey literature, providing a comprehensive overview of the methodological approaches for reporting surgical volume. The inclusion of grey literature was an important strength because it captured national reports, policy documents and health information system guidance that are often unavailable in peer-reviewed publications. The broad search strategy across multiple bibliographic databases and grey literature sources enabled the identification of diverse data collection systems and reporting practices from a wide range of healthcare settings. The use of a predefined protocol and standardised data extraction process further enhanced the transparency and reproducibility of the review.

This review has a few limitations. First, despite a comprehensive search of bibliographic databases and grey literature sources, some relevant reports or institutional documents may not have been publicly available or indexed and therefore were not have been identified. Second, the review was limited to studies published in English, which may have resulted in the omission of relevant evidence published in other languages. Third, patients and members of the public were not involved in the design, conduct or reporting of this review. Although this is common for evidence synthesis studies, stakeholder involvement may have provided additional perspectives on the practical implementation and use of surgical volume data collection systems. Finally, although the review followed a pre-defined protocol, it was neither publicly registered nor published, which may limit transparency regarding the planned review methods.

## Conclusion

Accurate measurement of surgical volume depends not only on collecting data, but also on how surgeries are defined, coded, validated and reported. This scoping review identified substantial methodological variation in approaches to estimating and reporting surgical volume. It highlights important gaps in data sources, reporting practices, validation procedures and health system coverage. These findings underscore the need for standardised methodologies that incorporate digital health information systems, uniform definitions and coding practices, transparent reporting of data quality and comprehensive inclusion of surgical activity across all healthcare sectors. Incorporating data from large-scale surveys like Demographic Health Surveys (DHS) and administrative platforms such as HIMS can enhance the consistency and reliability of surgical volume data, especially in LMICs. Future research should focus on developing and evaluating standardised, prospective frameworks for measuring surgical volume to support monitoring of surgical system performance and inform evidence-based policy.

## Supplementary material

10.1136/bmjopen-2026-118534 online supplemental file 1

10.1136/bmjopen-2026-118534 online supplemental file 2 online supplemental file 2

10.1136/bmjopen-2026-118534 online supplemental file 3

10.1136/bmjopen-2026-118534 online supplemental file 4

## Data Availability

Data are available upon reasonable request.
