# Supplementary material for: Documenting lancet core surgical indicator on volumes: a scoping review of data collection methods
Source: BMJ Open. 2026 Nov 2;16(11):e118534. doi: 10.1136/bmjopen-2026-118534 (PMC13735403; doi:10.1136/bmjopen-2026-118534)
Supplement: Supplementary file 1 [file bmjopen-16-11-s002.pdf]

Documenting Lancet Core Surgical Indicator on Volumes: A Scoping Review of Data Collection Methods

Supplementary Table 2: Methodological characteristics of original studies and grey literature sources included in the scoping review

Characteristics		Original articles (n=60)	Grey literature (n=22)	Total (N=82)
Coverage of Data				
	Nationally representative	40 (66.7%)	7 (31.8%)	47 (57.3%)
	Hospital-based	18 (30%)	6 (27.3%)	24 (28.3%)
	Consortium-based	2 (3.3%)	0 (0.0%)	2 (2.4%)
	Mixed / Multiple sources	NR	4 (18.2%)	4 (4.9%)
	International / Multi-country	NR	3 (13.6%)	3 (3.7%)
	Registry-based	NR	2 (9.1%)	2 (2.4%)
Data collection period				
	< 1 Year	18 (30%)	NR	18 (22%)
	1-2 Years	25 (41.7%)	NR	25 (30.5%)
	>2 Years	17 (28.3%)	NR	17 (20.7%)
	Not specified	0 (0.0%)	22 (100.0%)	22 (26.8%)
Data compiler				
	Trained researcher	22 (36.7%)	NR	22 (26.8%)
	Facility personnel	20 (33.3%)	NR	20 (24.4%)
	EMR Data	16 (26.7%)	NR	16 (19.5%)
	Government health authority	NR	10 (45.5%)	10 (12.2%)
	International organization	NR	3 (13.6%)	3 (3.7%)
	Academic / Research institution	NR	2 (9.1%)	2 (2.4%)
	Professional society / Clinical registry	NR	2 (9.1%)	2 (2.4%)
	Multi-institutional collaboration	NR	2 (9.1%)	2 (2.4%)
	Other	NR	3 (13.6%)	3 (3.7%)
	Not available	2 (3.3%)	0 (0.0%)	2 (2.4%)
Type of procedures tracked				
	All surgeries	34 (56.7%)	15 (68.2%)	49 (59.8%)
	Specific surgeries	18 (30%)	3 (13.6%)	21 (25.6%)
	Emergency surgeries	4 (6.7%)	1 (4.5%)	5 (6.1%)
	Elective surgeries	3 (5.0%)	0 (0.0%)	3 (3.7%)
	Essential surgeries	1 (1.7%)	0 (0.0%)	1 (1.2%)
	Not reported	0 (0.0%)	3 (13.6%)	3 (3.7%)
Surgery coding mechanism				

Characteristics		Original articles (n=60)	Grey literature (n=22)	Total (N=82)
	International Classification of Diseases (ICD-9/10)	5 (8.3%)	5 (22.7%)	10 (12.2%)
	Japanese national insurance claim coding system	2 (3.3%)	0 (0.0%)	2 (2.4%)
	Current Procedural Terminology (CPT)	1 (1.7%)	0 (0.0%)	1 (1.2%)
	National Health Classification	0 (0.0%)	1 (4.5%)	1 (1.2%)
	NHS Clinical Coding	0 (0.0%)	1 (4.5%)	1 (1.2%)
	Other	0 (0.0%)	2 (9.1%)	2 (2.4%)
	Not specified	52 (86.7%)	13 (59.1%)	66 (79.3%)
Source of the data collection				
	Paper based hospital log books	34 (56.7%)	2 (9.1%)	36 (43.9%)
	Electronic Medical records	20 (33.3%)	9 (40.9%)	29 (35.4%)
	HMIS	2 (3.3%)	3 (13.6%)	5 (6.1%)
	Secondary data compilation / Synthesis	0 (0.0%)	4 (18.2%)	4 (4.9%)
	Hospital Survey	3 (5.0%)	0 (0.0%)	3 (3.7%)
	Registry-based data collection	0 (0.0%)	2 (9.1%)	2 (2.4%)
	Other	1 (1.7%)	1 (4.5%)	2 (2.4%)
	Not reported	0 (0.0%)	1 (4.5%)	1 (1.2%)
Definition of surgery				
	Utstein Definition	15 (25.0%)	6 (27.3%)	21 (25.6%)
	Procedure-specific definition	8 (13.3%)	3 (13.6%)	11 (13.4%)
	Surgery performed in operating theatre regardless of anaesthesia	9 (15.0%)	1 (4.5%)	10 (12.2%)
	Study-specific definition	3 (5.0%)	5 (22.7%)	8 (9.8%)
	No definition provided	25 (41.7%)	7 (31.8%)	32 (39.0%)
Definition of surgical volume				
	Number/rate of procedures per 100,000 population	23 (38.3%)	9 (40.9%)	32 (39.0%)
	Total number of specific operations/procedures without a population denominator	13 (21.7%)	1 (4.5%)	14 (17.1%)
	Total number of operations/procedures without a population denominator	12 (20.0%)	2 (9.1%)	14 (17.1%)
	Rate per 100,000 for a specific population/procedure	8 (13.3%)	2 (9.1%)	10 (12.2%)
	Alternative metrics such as person-time-adjusted surgical volume (tSV)	1 (1.7%)	0 (0.0%)	1 (1.2%)
	Surgical volume not explicitly defined	3 (5.0%)	8 (36.4%)	11 (13.4%)
Data Validation				
	Quality assurance procedures - Training, audits, double entry, photographs, re-abstraction, standardized tools	10 (16.7%)	7 (31.8%)	17 (20.7%)

Characteristics		Original articles (n=60)	Grey literature (n=22)	Total (N=82)
	Sampling/statistical validation - Bootstrap, sensitivity analysis, sampling validation, cross-validation of models	4 (6.7%)	0 (0.0%)	4 (4.9%)
	Gold-standard/independent verification - Prospective observation, independent reviewers, validation against complete annual datasets	3 (5.0%)	2 (9.1%)	5 (6.1%)
	No validation described	43 (71.7%)	13 (59.1%)	56 (68.3%)
Missing data handling				
	Exclusion of incomplete records/facilities	8 (13.3%)	0 (0.0%)	8 (9.8%)
	Alternative estimation/weighting	5 (8.3%)	1 (4.5%)	6 (7.3%)
	Multiple imputation	4 (6.7%)	1 (4.5%)	5 (6.1%)
	Missing data retained and reported	2 (3.3%)	1 (4.5%)	3 (3.7%)
	Data quality monitoring/documentation	1 (1.7%)	4 (18.2%)	5 (6.1%)
	No description of missing-data handling	40 (66.7%)	15 (68.2%)	55 (67.1%)
Key methodological limitations*				
	Limitation explicitly relates to retrospective use of routinely recorded data.	51 (85.0%)	NR	51 (62.2%)
	Explicit concern that findings may not apply to a wider population, setting, region, or health system.	39 (65.0%)	NR	39 (47.6%)
	Missing records, missing months, incomplete documentation, or incomplete data are identified as a limitation.	33 (55.0%)	NR	33 (40.2%)
	Coding errors, transcription errors, misclassification, observer bias, or recording bias are identified.	27 (45.0%)	NR	27 (32.9%)
	Only selected specialties, procedures, subgroups, acuity groups, or settings are captured.	26 (43.3%)	NR	26 (31.7%)
	Manual, paper, handwritten logbooks or registers are identified as a limitation.	23 (38.3%)	NR	23 (28.0%)
	Some facilities were not included, not functional, non-participating, inaccessible, or refused data sharing.	22 (36.7%)	NR	22 (26.8%)
	Study is restricted to one centre, a small number of centres, or a limited geographical area.	21 (35.0%)	NR	21 (25.6%)
	Limited patient-level, clinical, outcome, risk-adjustment, or contextual variables are noted.	20 (33.3%)	NR	20 (24.4%)
	Modelled estimates, weighting, extrapolation, annualization, or related assumptions are identified as a limitation.	19 (31.7%)	NR	19 (23.2%)
	Data explicitly cover only public facilities or exclude private-sector activity.	16 (26.7%)	NR	16 (19.5%)

Characteristics		Original articles (n=60)	Grey literature (n=22)	Total (N=82)
	Study uses a short period, sampled months/days, or annualization/extrapolation from incomplete time coverage.	16 (26.7%)	NR	16 (19.5%)
	Procedures outside operating theatres/procedure areas are explicitly excluded or not captured.	5 (8.3%)	NR	5 (6.1%)
	Limited geographic / national coverage	NR	10 (45.5%)	10 (12.2%)
	Secondary data / administrative database	NR	8 (36.4%)	8 (9.8%)
	Lack of methodological transparency	NR	6 (27.3%)	6 (7.3%)
	Limited surgical scope	NR	5 (22.7%)	5 (6.1%)
	Heterogeneity / lack of standardization	NR	5 (22.7%)	5 (6.1%)
	Weak data collection system / routine reporting	NR	4 (18.2%)	4 (4.9%)
	Incomplete / missing data	NR	3 (13.6%)	3 (3.7%)
	Lack of validation / quality assessment	NR	3 (13.6%)	3 (3.7%)
	Limited clinical detail	NR	3 (13.6%)	3 (3.7%)
	Resource / system constraints	NR	3 (13.6%)	3 (3.7%)
	Modelling / proxy estimation	NR	1 (4.5%)	1 (1.2%)
	Limited generalizability	NR	1 (4.5%)	1 (1.2%)

*NR: Not Reported; * Categories are not mutually exclusive. A single study/grey literature resource may report multiple limitations; therefore, total percentages may exceed 100%; Utstein Definition: Incision, excision, or manipulation of tissue requiring regional/general anaesthesia or profound sedation performed in operating theatre*
