# Supplementary material for: Documenting lancet core surgical indicator on volumes: a scoping review of data collection methods
Source: BMJ Open. 2026 Nov 2;16(11):e118534. doi: 10.1136/bmjopen-2026-118534 (PMC13735403; doi:10.1136/bmjopen-2026-118534)
Supplement: Supplementary file 2 [file bmjopen-16-11-s003.pdf]

Documenting Lancet Core Surgical Indicator on Volumes: A Scoping Review of Data Collection Methods

Supplementary Table 3: Detailed data extraction of 60 original (peer-reviewed) articles

No	Author, year, country	Study setting	Health system setting	Definition of surgery	Definition of surgical volume	Data source	Data collection type	Procedure types tracked	Validation	Missing data handling
1	Weeks WB et al., 2014, France, US & Britain [28]	Rural + Urban	Public + Private	No definition provided	Surgical volume not explicitly defined	EMR	Retrospective	Elective surgeries	No validation described	Not mentioned
2	Bjerring AW et al., 2015, Sierra Leone [29]	Rural + Urban	Public + Private	Utstein Definition	Number/rate of procedures per 100,000 population	OT Logbooks	Retrospective	All surgeries	No validation described	Exclusion of incomplete records/facilities
3	Ross SW et al., 2022, USA [30]	Urban	Public	No definition provided	Total number of operations/procedures without a population denominator	EMR	Retrospective	Emergency surgeries	No validation described	Exclusion of incomplete records/facilities
4	Stokes MA et al., 2017, Papua New Guinea [31]	Rural	Public	Surgery performed in operating theatre regardless of anaesthesia	Number/rate of procedures per 100,000 population	OT Logbooks	Prospective	Specific surgeries	Sampling/statistical validation - Bootstrap, sensitivity analysis, sampling validation, cross-validation of models	Not mentioned

No	Author, year, country	Study setting	Health system setting	Definition of surgery	Definition of surgical volume	Data source	Data collection type	Procedure types tracked	Validation	Missing data handling
5	Gyedu A, 2020, Ghana [32]	Rural + Urban	Public	Surgery performed in operating theatre regardless of anaesthesia	Rate per 100,000 for a specific population/procedure	OT Logbooks	Retrospective	Specific surgeries	No validation described	Not mentioned
6	Chichom-Mefire A et al., 2017, Cameroon [33]	Rural + Urban	Public + Private	Study-specific definition	Number/rate of procedures per 100,000 population	OT Logbooks	Retrospective	All surgeries	Quality assurance procedures - Training, audits, double entry, photographs, re-abstraction, standardized tools	Not mentioned
7	Burgos CM et al., 2018, Sierra Leone [34]	Rural + Urban	Public + Private	Procedure-specific definition	Rate per 100,000 for a specific population/procedure	OT Logbooks	Retrospective	Specific surgeries	No validation described	Missing data retained and reported
8	Gyedu A et al., 2019, Ghana [35]	Rural + Urban	Public	No definition provided	Rate per 100,000 for a specific population/procedure	OT Logbooks	Retrospective	All surgeries	No validation described	Exclusion of incomplete records/facilities

No	Author, year, country	Study setting	Health system setting	Definition of surgery	Definition of surgical volume	Data source	Data collection type	Procedure types tracked	Validation	Missing data handling
9	Kakembo N et al., 2023, Uganda [36]	Rural + Urban	Public	No definition provided	Rate per 100,000 for a specific population/procedure	EMR	Prospective	All surgeries	No validation described	Exclusion of incomplete records/facilities
10	Gyedu A et al., 2020, Ghana [37]	Rural + Urban	Public	Surgery performed in operating theatre regardless of anaesthesia	Rate per 100,000 for a specific population/procedure	OT Logbooks	Retrospective	All surgeries	No validation described	Not mentioned
11	Cossa M et al., 2021, Mozambique [38]	Rural + Urban	Public	Surgery performed in operating theatre regardless of anaesthesia	Number/rate of procedures per 100,000 population	OT Logbooks	Prospective	All surgeries	No validation described	Not mentioned
12	Albutt K et al., 2019, Uganda [39]	Rural + Urban	Public	Utstein Definition	Number/rate of procedures per 100,000 population	OT Logbooks	Retrospective	All surgeries	Gold-standard/independent verification - Prospective observation, independent reviewers, validation against complete	Not mentioned

No	Author, year, country	Study setting	Health system setting	Definition of surgery	Definition of surgical volume	Data source	Data collection type	Procedure types tracked	Validation	Missing data handling
									annual datasets	
13	Weiser TG et al., 2016, 194 member states of the WHO [40]	Urban	Public + Private	Utstein Definition	Number/rate of procedures per 100,000 population	OT Logbooks	Retrospective	All surgeries	Sampling/statistical validation - Bootstrap, sensitivity analysis, sampling validation, cross-validation of models	Multiple imputation
14	Liu I et al., 2023, United States [41]	Rural + Urban	Public + Private	No definition provided	Total number of specific operations/procedures without a population denominator	EMR	Retrospective	Specific surgeries	No validation described	Not mentioned
15	Bolkan HA et al., 2015, Sierra Leone [42]	Rural + Urban	Public + Private	Utstein Definition	Number/rate of procedures per 100,000 population	OT Logbooks	Retrospective	All surgeries	Quality assurance procedures - Training, audits, double entry, photographs, re-abstraction,	Missing data retained and reported

No	Author, year, country	Study setting	Health system setting	Definition of surgery	Definition of surgical volume	Data source	Data collection type	Procedure types tracked	Validation	Missing data handling
									standardized tools	
16	Bhandarkar P et al., 2021, India [43]	Urban	Public	Study-specific definition	Number/rate of procedures per 100,000 population	EMR	Retrospective	All surgeries	No validation described	Not mentioned
17	O'Reilly-Shah et al., 2020, WHO regions [44]	Rural + Urban	Public + Private	No definition provided	Surgical volume not explicitly defined	OT Logbooks	Retrospective	All surgeries	No validation described	Exclusion of incomplete records/facilities
18	Gyedu A et al., 2018, Ghana [45]	Rural + Urban	Public	Surgery performed in operating theatre regardless of anaesthesia	Number/rate of procedures per 100,000 population	OT Logbooks	Retrospective	All surgeries	No validation described	Not mentioned
19	Shukla D et al., 2021, USA [46]	Urban	Private	No definition provided	Total number of operations/procedures without a population denominator	EMR	Retrospective	Elective surgeries	No validation described	Alternative estimation/weighting
20	Rajaguru P et al., 2019, Tanzania [47]	Rural	Public	No definition provided	Total number of operations/procedures without a population denominator	OT Logbooks	Retrospective	All surgeries	Gold-standard/independent verification - Prospective observation, independent reviewers,	Exclusion of incomplete records/facilities

No	Author, year, country	Study setting	Health system setting	Definition of surgery	Definition of surgical volume	Data source	Data collection type	Procedure types tracked	Validation	Missing data handling
									validation against complete annual datasets	
21	Hervochon R et al., 2020, France [48]	Rural	Public	Utstein Definition	Total number of operations/procedures without a population denominator	EMR	Retrospective	Specific surgeries	No validation described	Not mentioned
22	Adde HA et al., 2022, Liberia [49]	Rural + Urban	Public + Private	Utstein Definition	Total number of operations/procedures without a population denominator	OT Logbooks	Retrospective	All surgeries	Sampling/statistical validation - Bootstrap, sensitivity analysis, sampling validation, cross-validation of models	Exclusion of incomplete records/facilities
23	Pérez-Soto RH et al., 2023, Mexico [50]	Rural + Urban	Public	No definition provided	Number/rate of procedures per 100,000 population	EMR	Retrospective	All surgeries	No validation described	Not mentioned
24	Gyedu A et al., 2020, Ghana [51]	Rural + Urban	Public	Surgery performed in operating theatre	Rate per 100,000 for a specific	OT Logbooks	Retrospective	Specific surgeries	Sampling/statistical validation - Bootstrap,	Alternative estimation/weighting

No	Author, year, country	Study setting	Health system setting	Definition of surgery	Definition of surgical volume	Data source	Data collection type	Procedure types tracked	Validation	Missing data handling
				regardless of anaesthesia	population/procedure				sensitivity analysis, sampling validation, cross-validation of models	
25	Butler EK et al., 2021, Ghana [52]	Rural + Urban	Public	Surgery performed in operating theatre regardless of anaesthesia	Rate per 100,000 for a specific population/procedure	OT Logbooks	Retrospective	Emergency surgeries	Quality assurance procedures - Training, audits, double entry, photographs, re-abstraction, standardized tools	Alternative estimation/weighting
26	Eton RE et al., 2023, United States [53]	Urban	Public + Private	No definition provided	Total number of operations/procedures without a population denominator	EMR	Retrospective	Specific surgeries	Quality assurance procedures - Training, audits, double entry, photographs, re-abstraction, standardized tools	Not mentioned

No	Author, year, country	Study setting	Health system setting	Definition of surgery	Definition of surgical volume	Data source	Data collection type	Procedure types tracked	Validation	Missing data handling
27	Uribe-Leitz T et al., 2015, Global (21 GBD regions) [54]	Rural + Urban	Public + Private	No definition provided	Number/rate of procedures per 100,000 population	OT Logbooks	Retrospective	All surgeries	No validation described	Multiple imputation
28	Lindheim-Minde B et al., 2021, Sierra Leone [55]	Rural + Urban	Public + Private	Utstein Definition	Number/rate of procedures per 100,000 population	OT Logbooks	Retrospective	All surgeries	Quality assurance procedures - Training, audits, double entry, photographs, re-abstraction, standardized tools	Alternative estimation/weighting
29	De Plecker E et al., 2017, Burundi [56]	Rural	Public	No definition provided	Total number of specific operations/procedures without a population denominator	OT Logbooks	Retrospective	Emergency surgeries	Quality assurance procedures - Training, audits, double entry, photographs, re-abstraction, standardized tools	Not mentioned

No	Author, year, country	Study setting	Health system setting	Definition of surgery	Definition of surgical volume	Data source	Data collection type	Procedure types tracked	Validation	Missing data handling
30	Massenburg BB et al., 2017, Brazil [57]	Rural + Urban	Public	No definition provided	Number/rate of procedures per 100,000 population	EMR	Retrospective	All surgeries	No validation described	Not mentioned
31	Cheelo M et al., 2018, Zambia [58]	Rural	Public	No definition provided	Total number of specific operations/procedures without a population denominator	OT Logbooks	Prospective	Specific surgeries	No validation described	Not mentioned
32	Tefera A et al., 2019, KwaZulu-Natal Province [59]	Rural + Urban	Public	Surgery performed in operating theatre regardless of anaesthesia	Total number of operations/procedures without a population denominator	OT Logbooks	Retrospective	All surgeries	No validation described	Not mentioned
33	Anderson JE et al., 2019, Nigeria [60]	Rural + Urban	Public	No definition provided	Number/rate of procedures per 100,000 population	Hospital Survey	Retrospective	All surgeries	No validation described	Not mentioned
34	Joharifard S et al., 2020, Liberia [61]	Rural	Public	No definition provided	Total number of operations/procedures without a population denominator	OT Logbooks	Retrospective	All surgeries	No validation described	Not mentioned
35	Löfgren J et al., 2015, Uganda [62]	Rural	Public	Utstein Definition	Number/rate of procedures per 100,000 population	OT Logbooks	Prospective	Specific surgeries	Quality assurance procedures - Training,	Not mentioned

No	Author, year, country	Study setting	Health system setting	Definition of surgery	Definition of surgical volume	Data source	Data collection type	Procedure types tracked	Validation	Missing data handling
									audits, double entry, photographs, re-abstraction, standardized tools	
36	Sani R et al., 2015, Niger [63]	Rural	Public	No definition provided	Total number of operations/procedures without a population denominator	OT Logbooks	Prospective	All surgeries	No validation described	Not mentioned
37	Ajiko M et al., 2017, Uganda [64]	Rural	Public	No definition provided	Total number of operations/procedures without a population denominator	OT Logbooks	Retrospective	All surgeries	No validation described	Not mentioned
38	Albutt K et al., 2019, Uganda [65]	Rural + Urban	Private	Utstein Definition	Total number of operations/procedures without a population denominator	OT Logbooks	Retrospective	All surgeries	No validation described	Not mentioned
39	Bolkan HA et al., 2018, Sierra Leone [66]	Rural + Urban	Public	Utstein Definition	Total number of operations/procedures without a population denominator	OT Logbooks	Retrospective	All surgeries	No validation described	Not mentioned

No	Author, year, country	Study setting	Health system setting	Definition of surgery	Definition of surgical volume	Data source	Data collection type	Procedure types tracked	Validation	Missing data handling
40	Albutt K, 2018, Uganda [67]	Rural + Urban	Public	Utstein Definition	Number/rate of procedures per 100,000 population	OT Logbooks	Retrospective	All surgeries	No validation described	Not mentioned
41	Siddiqi S, 2020, Pakistan [68]	Rural	Public	No definition provided	Number/rate of procedures per 100,000 population	Hospital Survey	Retrospective	Essential surgeries	No validation described	Not mentioned
42	Concepcion T L, 2019, Somaliland [69]	Rural + Urban	Public + Private	No definition provided	Total number of specific operations/procedures without a population denominator	OT Logbooks	Retrospective	Specific surgeries	No validation described	Multiple imputation
43	Anderson GA, 2017, Uganda [70]	Urban	Public	Surgery performed in operating theatre regardless of anaesthesia	Number/rate of procedures per 100,000 population	OT Logbooks	Retrospective	All surgeries	Gold-standard/independent verification - Prospective observation, independent reviewers, validation against complete annual datasets	Not mentioned

No	Author, year, country	Study setting	Health system setting	Definition of surgery	Definition of surgical volume	Data source	Data collection type	Procedure types tracked	Validation	Missing data handling
44	Sankar A, et.al, 2024, Canada [71]	Rural + Urban	Public	Procedure-specific definition	Number/rate of procedures per 100,000 population	EMR	Retrospective	Specific surgeries	No validation described	Not mentioned
45	Anand E et.al, 2024, United Kingdom [72]	Rural + Urban	Public	Procedure-specific definition	Total number of specific operations/procedures without a population denominator	EMR	Prospective	Emergency surgeries	No validation described	Multiple imputation
46	Alsakarneh S et.al, 2024, USA [73]	Rural + Urban	Public + Private	No definition provided	Total number of specific operations/procedures without a population denominator	EMR	Retrospective	Specific surgeries	No validation described	Not mentioned
47	Yoneda H et.al, 2025, Japan [74]	Rural + Urban	Public + Private	Procedure-specific definition	Total number of specific operations/procedures without a population denominator	EMR	Retrospective	All surgeries	No validation described	Not mentioned
48	Junqueira BL et.al, 2024, USA [75]	Urban	Public	Procedure-specific definition	Total number of specific operations/procedures without a population denominator	EMR	Retrospective	All surgeries	No validation described	Not mentioned

No	Author, year, country	Study setting	Health system setting	Definition of surgery	Definition of surgical volume	Data source	Data collection type	Procedure types tracked	Validation	Missing data handling
49	Abid M et.al, 2026, North Carolina [76]	Rural + Urban	Public + Private	No definition provided	Surgical volume not explicitly defined	EMR	Retrospective	Specific surgeries	No validation described	Not mentioned
50	Furre ME et.al, 2025, Sierra Leone [77]	Rural + Urban	Public + Private	Utstein Definition	Number/rate of procedures per 100,000 population	OT Logbooks	Retrospective	All surgeries	Quality assurance procedures - Training, audits, double entry, photographs, re-abstraction, standardized tools	Alternative estimation/weighting
51	Asano S et.al, 2025, Japan [78]	Rural + Urban	Public + Private	No definition provided	Alternative metrics such as person-time-adjusted surgical volume (tSV)	EMR	Retrospective	Specific surgeries	No validation described	Not mentioned
52	Bittencourt PL et.al, 2024, Brazil [79]	Urban	Private	Procedure-specific definition	Total number of specific operations/procedures without a population denominator	EMR	Retrospective	Specific surgeries	No validation described	Not mentioned
53	Fowler XP et.al, 2024, USA [80]	Rural + Urban	Public + Private	No definition provided	Total number of specific operations/procedures without a	EMR	Retrospective	Specific surgeries	No validation described	Not mentioned

No	Author, year, country	Study setting	Health system setting	Definition of surgery	Definition of surgical volume	Data source	Data collection type	Procedure types tracked	Validation	Missing data handling
					population denominator					
54	Zadey S et.al, 2024, India [21]	Rural + Urban	Public + Private	Utstein Definition	Number/rate of procedures per 100,000 population	HMIS	Retrospective	All surgeries	No validation described	Not mentioned
55	Martens JPJ et.al, 2025, Sierra Leone [81]	Rural + Urban	Public + Private	Utstein Definition	Number/rate of procedures per 100,000 population	OT Logbooks	Retrospective	All surgeries	Quality assurance procedures - Training, audits, double entry, photographs, re-abstraction, standardized tools	Not mentioned
56	Siddiqi S, et.al, 2025, Pakistan [82]	Rural	Public	No definition provided	Rate per 100,000 for a specific population/procedure	Hospital Survey	Retrospective	Specific surgeries	No validation described	Not mentioned
57	Shabani J, 2023, Tanzania (Mainland) [83]	Rural + Urban	Public + Private	Procedure-specific definition	Total number of specific operations/procedures without a population denominator	HMIS	Retrospective	Specific surgeries	No validation described	Not mentioned

No	Author, year, country	Study setting	Health system setting	Definition of surgery	Definition of surgical volume	Data source	Data collection type	Procedure types tracked	Validation	Missing data handling
58	Rukhsar A, 2025, India [84]	Urban	Public	Study-specific definition	Total number of specific operations/procedures without a population denominator	EMR	Retrospective	Elective surgeries	No validation described	Not mentioned
59	Cook K, 2024, Ethiopia [85]	Rural + Urban	Public	Utstein Definition	Number/rate of procedures per 100,000 population	OT Logbooks | EMR	Retrospective	All surgeries	Quality assurance procedures - Training, audits, double entry, photographs, re-abstraction, standardized tools	Data quality monitoring/documentation
60	Matousek A, 2015, Haiti [86]	Rural	Public	Procedure-specific definition	Total number of specific operations/procedures without a population denominator	OT Logbooks	Retrospective	All surgeries	No validation described	Exclusion of incomplete records/facilities

Utstein Definition: Incision, excision, or manipulation of tissue requiring regional/general anesthesia or profound sedation performed in the operating theatre; EMR: Electronic Medical Record; OT Logbooks: Paper-based hospital logbooks

References:

21. Zadey S, Smith ER, Staton CA, Fitzgerald TN, Vissoci JRN. Population-level surgical rates and unmet need in India: a retrospective analysis of districts and states from 2011 to 2019. *International Journal of Surgery*. 2024;110(3):1884-1887. doi:10.1097/JS9.0000000000001024

28. Weeks WB, Paraponaris A, Ventelou B. Geographic variation in rates of common surgical procedures in France in 2008–2010, and comparison to the US and Britain. *Health Policy*. 2014;118(2):215-221. doi:10.1016/j.healthpol.2014.08.015

29. Bjerring AW, Lier ME, Rød SM, et al. Assessing cesarean section and inguinal hernia repair as proxy indicators of the total number of surgeries performed in Sierra Leone in 2012. *Surgery*. 2015;157(5):836-842. doi:10.1016/j.surg.2014.12.014

30. Ross SW, McCartt JC, Cunningham KW, et al. Emergencies do not shut down during a pandemic: COVID pandemic impact on Acute Care Surgery volume and mortality at a level I trauma center. *The American Journal of Surgery*. 2022;224(6):1409-1416. doi:10.1016/j.amjsurg.2022.10.030

31. Stokes MAR, Guest GD, Mamadi P, et al. Measuring the Burden of Surgical Disease Averted by Emergency and Essential Surgical Care in a District Hospital in Papua New Guinea. *World j surg*. 2017;41(3):650-659. doi:10.1007/s00268-016-3769-6

32. Gyedu A, Stewart B, Gaskill C, Donkor P, Quansah R, Mock C. Benchmarking Global Trauma Care: Defining the Unmet Need for Trauma Surgery in Ghana. *Journal of Surgical Research*. 2020;247:280-286. doi:10.1016/j.jss.2019.10.013

33. Chichom-Mefire A, Mbome Njie V, Verla V, Atashili J. A Retrospective One-Year Estimation of the Volume and Nature of Surgical and Anaesthetic Services Delivered to the Populations of the Fako Division of the South-West Region of Cameroon: An Urgent Call for Action. *World j surg*. 2017;41(3):660-671. doi:10.1007/s00268-016-3775-8

34. Burgos CM, Bolkan HA, Bash-Taqi D, Hagander L, Von Screeb J. The Met Needs for Pediatric Surgical Conditions in Sierra Leone: Estimating the Gap. *World j surg*. 2018;42(3):652-665. doi:10.1007/s00268-017-4244-8

35. Gyedu A, Stewart B, Gaskill C, et al. Enumeration of Operations Performed for Elderly Patients in Ghana: An Opportunity to Improve Global Surgery Benchmarking. *World j surg*. 2019;43(7):1644-1652. doi:10.1007/s00268-019-04963-7

36. Kakembo N, Grabski DF, Situma M, et al. Met and Unmet Need for Pediatric Surgical Access in Uganda: A Country-Wide Prospective Analysis. *Journal of Surgical Research*. 2023;286:23-34. doi:10.1016/j.jss.2022.12.036

37. Gyedu A, Stewart B, Wadie R, Antwi J, Donkor P, Mock C. Population-based rates of hernia surgery in Ghana. *Hernia*. 2020;24(3):617-623. doi:10.1007/s10029-019-02027-2

38. Cossa M, Rose J, Berndtson AE, Noormahomed E, Bickler SW. Assessment of Surgical Care Provided in National Health Services Hospitals in Mozambique: The Importance of Subnational Metrics in Global Surgery. *World j surg*. 2021;45(5):1306-1315. doi:10.1007/s00268-020-05925-0

39. Albutt K, Punchak M, Kayima P, Namanya DB, Shrime MG. Operative volume and surgical case distribution in Uganda's public sector: a stratified randomized evaluation of nationwide surgical capacity. *BMC Health Serv Res*. 2019;19(1):104. doi:10.1186/s12913-019-3920-9

40. Weiser TG, Haynes AB, Molina G, et al. Size and distribution of the global volume of surgery in 2012. *Bull World Health Organ*. 2016;94(3):201-209F. doi:10.2471/BLT.15.159293

41. Liu IZ, Whitehead J, Schlafly M, Pakhchanian H, Raiker R, Snoddy MC. Quantifying the Impact of COVID-19 on Hand and Wrist Surgery Procedural Volume: A National Analysis of 381,046 Cases. *Journal of Hand Surgery Global Online*. 2023;5(2):151-158. doi:10.1016/j.jhsg.2022.12.004

42. Bolkan HA, Von Schreeb J, Samai MM, et al. Met and unmet needs for surgery in Sierra Leone: A comprehensive, retrospective, countrywide survey from all health care facilities performing operations in 2012. *Surgery*. 2015;157(6):992-1001. doi:10.1016/j.surg.2014.12.028

43. Bhandarkar P, Gadgil A, Patil P, Mohan M, Roy N. Estimation of the National Surgical Needs in India by Enumerating the Surgical Procedures in an Urban Community Under Universal Health Coverage. *World j surg*. 2021;45(1):33-40. doi:10.1007/s00268-020-05794-7

44. O'Reilly-Shah VN, Van Cleve W, Long DR, et al. Impact of COVID-19 response on global surgical volumes: an ongoing observational study. *Bull World Health Organ*. 2020;98(10):671-682. doi:10.2471/BLT.20.264044

45. Gyedu A, Stewart B, Gaskill C, et al. Improving Benchmarks for Global Surgery: Nationwide Enumeration of Operations Performed in Ghana. *Annals of Surgery*. 2018;268(2):282-288. doi:10.1097/SLA.0000000000002457

46. Shukla D, Patel S, Clack L, Smith TB, Shuler MS. Retrospective analysis of trends in surgery volumes between 2016 and 2019 and impact of the insurance deductible: Cross-sectional study. *Annals of Medicine & Surgery*. 2021;63. doi:10.1016/j.amsu.2021.02.022

47. Rajaguru PP, Jusabani MA, Massawe H, Temu R, Sheth NP. Understanding surgical care delivery in Sub-Saharan Africa: a cross-sectional analysis of surgical volume, operations, and financing at a tertiary referral hospital in rural Tanzania. *glob health res policy*. 2019;4(1):30. doi:10.1186/s41256-019-0122-2

48. Hervochon R, Atallah S, Levivien S, Teissier N, Baujat B, Tankere F. Impact of the COVID-19 epidemic on ENT surgical volume. *European Annals of Otorhinolaryngology, Head and Neck Diseases*. 2020;137(4):269-271. doi:10.1016/j.anorl.2020.08.006

49. Adde HA, Van Duinen AJ, Sherman LM, et al. A Nationwide Enumeration of the Surgical Workforce, its Production and Disparities in Operative Productivity in Liberia. *World j surg*. 2022;46(3):486-496. doi:10.1007/s00268-021-06379-8

50. Pérez-Soto RH, Trolle-Silva AM, Valdés GABR, et al. Timely Access to Essential Surgery, Surgical Workforce, and Surgical Volume: Global Surgery Indicators in Mexico. *Glob Health Sci Pract*. 2023;11(1):e2100745. doi:10.9745/GHSP-D-21-00745

51. Gyedu A, Lester L, Stewart B, et al. Estimating obstetric and gynecologic surgical rate: A benchmark of surgical capacity building in Ghana. *Intl J Gynecology & Obste*. 2020;148(2):205-209. doi:10.1002/ijgo.13019

52. Butler EK, Gyedu A, Stewart BT, Quansah R, Donkor P, Mock CN. Nationwide enumeration of emergency operations performed in Ghana. *Eur J Trauma Emerg Surg*. 2021;47(4):1031-1039. doi:10.1007/s00068-019-01276-1

53. Eton RE, Yost ML, Thompson MP, et al. Association of Elective Surgical Volume With State Executive Order Curtailing Elective Surgery in Michigan During the COVID-19 Pandemic. *Annals of Surgery*. 2023;277(2):223-227. doi:10.1097/SLA.0000000000005150

54. Uribe-Leitz T, Esquivel MM, Molina G, et al. Projections for Achieving the Lancet Commission Recommended Surgical Rate of 5000 Operations per 100,000 Population by Region-Specific Surgical Rate Estimates. *World j surg*. 2015;39(9):2168-2172. doi:10.1007/s00268-015-3113-6

55. Lindheim-Minde B, Gjøra A, Bakker JM, et al. Changes in surgical volume, workforce, and productivity in Sierra Leone between 2012 and 2017. *Surgery*. 2021;170(1):126-133. doi:10.1016/j.surg.2021.02.043

56. De Plecker E, Zachariah R, Kumar AMV, et al. Emergency obstetric care in a rural district of Burundi: What are the surgical needs? Puebla I, ed. *PLoS ONE*. 2017;12(2):e0170882. doi:10.1371/journal.pone.0170882

57. Massenburg BB, Saluja S, Jenny HE, et al. Assessing the Brazilian surgical system with six surgical indicators: a descriptive and modelling study. *BMJ Glob Health*. 2017;2(2):e000226. doi:10.1136/bmjgh-2016-000226

58. Cheelo M, Brugha R, Bijlmakers L, Kachimba J, McCauley T, Gajewski J. Surgical Capacity at District Hospitals in Zambia: From 2012 to 2016. *World j surg*. 2018;42(11):3508-3513. doi:10.1007/s00268-018-4678-7

59. Tefera A, Lutge E, Sartorius B, Clarke D. The Operative Output of District Hospitals in KwaZulu-Natal Province is Heavily Skewed Toward Obstetrical Care. *World j surg*. 2019;43(7):1653-1660. doi:10.1007/s00268-019-04985-1

60. Anderson JE, Ndajiwo AB, Nuhu SA, Lawal OA, Amedu JO, Ameh EA. Assessment of Capacity to Meet Lancet Commission on Global Surgery Indicators in the Federal Capital Territory, Abuja, Nigeria. *World j surg*. 2019;43(3):704-714. doi:10.1007/s00268-018-4835-z

61. Joharifard S, Price N, Ipanaqué JGY, et al. The Impact of Implementing a Comprehensive Surgical Program on the Surgical Cohort at a Remote Referral Hospital in Southeastern Liberia. *World j surg*. 2020;44(3):680-688. doi:10.1007/s00268-019-05277-4

62. Löfgren J, Kadobera D, Forsberg BC, Mulowooza J, Wladis A, Nordin P. District-level surgery in Uganda: Indications, interventions and perioperative mortality. *Surgery*. 2015;158(1):7-16. doi:10.1016/j.surg.2015.03.022

63. Sani R, Sanoussi S, Didier JL, Salifou GM, Abarchi H. Rural Surgery in Niger: A Multicentric Study in 21 District Hospitals. *Indian J Surg*. 2015;77(S3):822-826. doi:10.1007/s12262-013-1015-0

64. Ajiko MM, Davé D, Feldhaus I, Smith RN, Juillard C, Dicker RA. Patterns of surgical presentation at an African regional referral hospital: surveillance as a step towards improving access to care. *Eur J Trauma Emerg Surg*. 2017;43(2):265-272. doi:10.1007/s00068-016-0644-y

65. Albutt K, Drevin G, Yorlets RR, et al. 'We are all serving the same Ugandans': A nationwide mixed-methods evaluation of private sector surgical capacity in Uganda. Torpey K, ed. *PLoS ONE*. 2019;14(10):e0224215. doi:10.1371/journal.pone.0224215

66. Bolkan HA, Van Duinen A, Samai M, et al. Admissions and surgery as indicators of hospital functions in Sierra Leone during the west-African Ebola outbreak. *BMC Health Serv Res*. 2018;18(1):846. doi:10.1186/s12913-018-3666-9

67. Albutt K, Punchak M, Kayima P, Namanya DB, Anderson GA, Shrime MG. Access to Safe, Timely, and Affordable Surgical Care in Uganda: A Stratified Randomized Evaluation of Nationwide Public Sector Surgical Capacity and Core Surgical Indicators. *World j surg*. 2018;42(8):2303-2313. doi:10.1007/s00268-018-4485-1

68. Siddiqi S, Khan MS, Rizvi N, et al. Are Rural Hospitals in Pakistan Responding to the Global Surgery Movement? An Analysis of the Gaps, Challenges and Opportunities. *World j surg*. 2020;44(4):1045-1052. doi:10.1007/s00268-019-05327-x

69. Concepcion TL, Smith ER, Mohamed M, et al. Provision of Surgical Care for Children Across Somaliland: Challenges and Policy Guidance. *World j surg*. 2019;43(11):2934-2944. doi:10.1007/s00268-019-05079-8

70. Anderson GA, Ilcisin L, Abesiga L, et al. Surgical volume and postoperative mortality rate at a referral hospital in Western Uganda: Measuring the Lancet Commission on Global Surgery indicators in low-resource settings. *Surgery*. 2017;161(6):1710-1719. doi:10.1016/j.surg.2017.01.009

71. Sankar A, Stukel TA, Baxter NN, et al. Disparities in surgery rates during the COVID-19 pandemic: retrospective study. *BJS Open*. 2024;8(4):zrae088. doi:10.1093/bjsopen/zrae088

72. Anand E, Rahman SA, Tomlinson C, Mercer SJ, Pucher PH. Comparison of major abdominal emergency surgery outcomes across organizational models of emergency surgical care: Analysis of the UK NELA national database. *J Trauma Acute Care Surg*. 2024;96(2):305-312. doi:10.1097/TA.0000000000004056

73. Alsakarneh S, Karna R, Shaukat A, Bilal M. Rates of colorectal surgery in patients with non-malignant colorectal polyps: Results from a nationwide study. *Endosc Int Open*. 2026;14(CP):a-2795-7563. doi:10.1055/a-2795-7563

74. Yoneda H, Curley J, Iwatsuki K, Saeki M, Takahashi N, Yamamoto M. Impact of COVID-19 Pandemic on Hand Surgery Volume in Japan. *JCM*. 2025;14(5):1518. doi:10.3390/jcm14051518

75. Junqueira BL, Taylor LM, Aston CE, Bewley B, Mason J, Rensing AJ. The effect of Covid-19 on pediatric surgical case volume. *Pediatric Anesthesia and Critical Care Journal*. 2024;(Vol.12, Issue 1):24-30. doi:10.14587/paccj.2024.4

76. Abid M, Holmes GM, Charles A. Measuring Surgical Access: The Correlation of Hospital and Patient-Focused Metrics. *Journal of Surgical Research*. 2026;321:491-499. doi:10.1016/j.jss.2026.03.018

77. Furre ME, Svengaard M, Øvreås E, et al. The impact of surgical task-sharing in Sierra Leone: a nationwide longitudinal observational study on surgical workforce and volume, 2012–2023. *BMJ Glob Health*. 2025;10(5):e018423. doi:10.1136/bmjgh-2024-018423

78. Asano S, Kunisawa S, Imanaka Y. Development of a new indicator of surgical burden using person-time-adjusted surgical volume: a cross-sectional regional analysis in Japan. *bmjph*. 2025;3(2):e002720. doi:10.1136/bmjph-2025-002720

79. Bittencourt PL, Aragão GVP, Valverde Filho MT, et al. Impact of COVID-19 pandemic on surgical volume and outcomes in a terciary care center in Brazil. *Rev Col Bras Cir*. 2024;51:e20243678. doi:10.1590/0100-6991e-20243678-en

80. Fowler XP, Krafcik B, Cai M, et al. A Retrospective Analysis of Surgical Acuity & Procedure Volume Before & During the COVID-19 Pandemic. *Journal of Surgical Research*. 2024;296:696-703. doi:10.1016/j.jss.2023.11.062

81. Martens JPJ, Van Leerdam D, Kanneh S, et al. Surgical Volume in Sierra Leone: A Comparison Between Population and Facility-Based Data Collection. *World j surg*. 2025;49(9):2523-2530. doi:10.1002/wjs.70050

82. Siddiqi S, Naeem I, Mehboob S, et al. Assessment of surgical services and needs in rural district and subdistrict hospitals in Pakistan. *East Mediterr Health J*. 2025;31(6):363-370. doi:10.26719/2025.31.6.363

83. Shabani J, Masanja H, Kagoye S, et al. Quality of reporting and trends of emergency obstetric and neonatal care indicators: an analysis from Tanzania district health information system data between 2016 and 2020. *BMC Pregnancy Childbirth*. 2023;23(1):716. doi:10.1186/s12884-023-06028-z

84. Rukhsar A, S SA, Sofi G. A Pattern of Surgical Procedures undertaken in the National Institute of Unani Medicine (NIUM) Hospital, Bengaluru, Karnataka, India: A Retrospective Study. *JOCAMR*. 2025;26(10). doi:https://doi.org/10.9734/jocamr/2025/v26i10721

85. Cook KR, Zeleke ZB, Gebrehana E, et al. Quality and sustainability of Ethiopia’s national surgical indicators. Alayande BT, ed. *PLOS Glob Public Health*. 2024;4(3):e0002600. doi:10.1371/journal.pgph.0002600

86. Matousek AC, Matousek SB, Addington SR, et al. The Struggle for Equity: An Examination of Surgical Services at Two NGO Hospitals in Rural Haiti. *World j surg*. 2015;39(9):2191-2197. doi:10.1007/s00268-015-3084-7
