# Supplementary material for: Documenting lancet core surgical indicator on volumes: a scoping review of data collection methods
Source: BMJ Open. 2026 Nov 2;16(11):e118534. doi: 10.1136/bmjopen-2026-118534 (PMC13735403; doi:10.1136/bmjopen-2026-118534)
Supplement: Supplementary file 3 [file bmjopen-16-11-s004.pdf]

Documenting Lancet Core Surgical Indicator on Volumes: A Scoping Review of Data Collection Methods

Supplementary Table 4: Detailed data extraction of 22 grey literature sources

No	Name of the report/website	Document Type	Country	Definition of surgery	Data source	Data collection type	Procedure types tracked	Data Validation	Missing data handling
1	Number of surgical procedures (per 100,000 population) [87]	Indicator metadata	Global	Utstein Definition	Secondary data compilation / Synthesis	Retrospective	All surgeries	No validation described	Not mentioned
2	Where are essential surgeries being performed and monitored? A global update on volume reporting [88]	Website	Global	Utstein Definition	Secondary data compilation / Synthesis	Retrospective	Not reported	No validation described	Missing data retained and reported
3	WHO European Health Information Gateway - Inpatient surgical procedures per year per 100,000 [89]	Website	Europe	Study-specific definition	Not reported	Not reported	Not reported	No validation described	Not mentioned
4	Global Surgery Metrics - Metadata for Global Surgery Indicators [90]	Indicator metadata	Global	Utstein Definition	Paper-based hospital records	Not reported	All surgeries	Gold-standard/independent verification	Multiple imputation
5	IFSO - 9th Global Registry Report [91]	Government/Technic	Global	Procedure-specific definition	Registry-based data collection	Both	Specific surgeries	Quality assurance procedures	Not mentioned

No	Name of the report/website	Document Type	Country	Definition of surgery	Data source	Data collection type	Procedure types tracked	Data Validation	Missing data handling
		al/Registry Report							
6	National Surgical, Obstetric & Anaesthesia Plan for Ghana [92]	National strategic plan	Ghana	Utstein Definition	OT Log Books	Retrospective	Specific surgeries	No validation described	Not mentioned
7	The Global Surgery Research Unit of the Latin American Indicator Research Collaboratory: An Action Model for Grassroots Collection of Lancet Commission on Global Surgery Indicators [93]	Government/Technical/Registry Report	Colombia (Medellín)	No definition provided	EMR	Retrospective	All surgeries	Gold-standard/independent verification	Not mentioned
8	Evaluation of Social Access to Safe Surgeries in the Philippines [94]	Government/Technical/Registry Report	Philippines	Surgery performed in operating theatre regardless of anaesthesia	Model-based estimation	Retrospective	All surgeries	No validation described	Alternative estimation/weighting
9	National Surgical, Obstetric, and Anesthesia Strategic Plan Development - The Zambian Experience [95]	National strategic plan	Zambia	No definition provided	Secondary data compilation / Synthesis	Retrospective	Not reported	No validation described	Not mentioned

No	Name of the report/website	Document Type	Country	Definition of surgery	Data source	Data collection type	Procedure types tracked	Data Validation	Missing data handling
10	A Comprehensive Framework for State-Level Health-System Design in India and a Case Study of Himachal Pradesh [96]	Government/Technical/Registry Report	India	No definition provided	Secondary data compilation / Synthesis	Retrospective	All surgeries	No validation described	Not mentioned
11	Health Information Management System (HIMS) [97]	Website	India	Utstein Definition	HMIS / Routine reporting systems	Prospective	All Surgeries	No validation described	Not mentioned
12	NHS Digital - Hospital Episode Statistics (HES) [98]	Website	England, UK	No definition provided	EMR	Prospective	All surgeries	Quality assurance procedures	Data quality monitoring/documentation
13	Electronic Health Solutions (Hakeem) [99]	Website	Jordan	No definition provided	EMR	Prospective	All surgeries	No validation described	Not mentioned
14	Medical Data Vision - Nippon Life Insurance Company Group [100]	Website	Japan	No definition provided	EMR	Prospective	All surgeries	No validation described	Not mentioned
15	Public Health Scotland - Scottish Morbidity Record [101]	Website	Scotland, UK	Study-specific definition	EmR	Prospective	All surgeries	Quality assurance procedures	Data quality monitoring/documentation

No	Name of the report/website	Document Type	Country	Definition of surgery	Data source	Data collection type	Procedure types tracked	Data Validation	Missing data handling
16	Research Data Assistance Center (ResDAC) [102]	Website	United states	Study-specific definition	EMR	Retrospective	All surgeries	No validation described	Not mentioned
17	Danish Health Data Authority [103]	Website	Denmark	Study-specific definition	EMR	Prospective	All surgeries	Quality assurance procedures	Not mentioned
18	Statewide Planning and Research Cooperative System (SPARCS) [104]	Website	United states	Study-specific definition	EMR	Prospective	All surgeries	Quality assurance procedures	Data quality monitoring/documentation
19	International Federation for Surgery and Other Therapies for Obesity [105]	Website	Multi-country	Procedure-specific definition	Registry-based data collection	Prospective	Specific surgeries	Quality assurance procedures	Not mentioned
20	Ministry of Health - Oman [106]	Website	Oman	Utstein Definition	HMIS / Routine reporting systems	Not reported	All surgeries	No validation described	Not mentioned
21	Rwanda Ministry of Health [107]	Website	Rwanda	No definition provided	HMIS / Routine reporting systems	Prospective	All surgeries	Quality assurance procedures	Not mentioned
22	National Emergency Laparotomy Audit (NELA) [108]	Website	England, Wales and	Procedure-specific definition	EMR	Prospective	Emergency surgeries	No validation described	Data quality monitorin

No	Name of the report/website	Document Type	Country	Definition of surgery	Data source	Data collection type	Procedure types tracked	Data Validation	Missing data handling
			Northern Ireland						g/documentation

Utstein Definition: Incision, excision, or manipulation of tissue requiring regional/general anesthesia or profound sedation performed in operating theatre; EMR: Electronic Medical Records; OT Logbooks: Paper-based hospital logbooks

References:

87. World Bank Open Data. Number of surgical procedures (per 100,000 population). Accessed July 26, 2026. https://data.worldbank.org/indicator/SH.SGR.PROC.P5

88. Davis G, Suzuki E, Rose J. Where are essential surgeries being performed and monitored? A global update on volume reporting. June 25, 2024. Accessed July 26, 2026. https://blogs.worldbank.org/en/opendata/where-are-essential-surgeries-being-performed-and-monitored--a-g

89. World Health Organization Regional Office for Europe. Data – European Health Information Gateway. Accessed July 26, 2026. https://gateway.euro.who.int/en/indicators/hfa_538-6030-inpatient-surgical-procedures-per-year-per-100-000/#id=19633

90. World Federation of Societies of Anaesthesiologists. Metadata for Global Surgery Indicators: Toolkit. 2025. https://wfsahq.org/resources/surgery-metrics/

91. Sakran N, Stenberg E, Batista C, Boza C. *International Federation for the Surgery of Obesity and Metabolic Disorders (IFSO). 9th Global Registry Report.* 2024. https://www.ifso.com/pdf/9th-ifso-global-registry-report-2024.pdf

92. *Ministry of Health. National Surgical, Obstetric and Anaesthesia Plan for Ghana (NSOAP) 2025–2029*. Ministry of Health; 2024. Accessed July 26, 2026. https://www.moh.gov.gh/wp-content/uploads/2024/10/FINAL-NSOAP-for-press-2.pdf

93. Ginalis EE, Morales C, Mendoza C, Hanna JS, Peck GL. The Global Surgery Research Unit of the Latin American Indicator Research Collaboratory: An Action Model for Grassroots Collection of Lancet Commission on Global Surgery Indicators. *In Review.* Preprint posted online May 5, 2021. doi:10.21203/rs.3.rs-476642/v1

94. Benedyk T. *Evaluation of Social Access to Safe Surgeries in the Philippines.* Cambridge: Polygeia Global Health Think Tank, University of Cambridge; 2021.
95. Mukhopadhyay S, Lin Y, Mwaba P, et al. Implementing World Health Assembly Resolution 68.15: National surgical, obstetric, and anesthesia strategic plan development—the Zambian experience. 2017;(6):23-31.
96. Mor N. A Comprehensive Framework for State-Level Health-System Design in India and a Case Study of Himachal Pradesh. Preprint posted online 2026.
97. Health Management Information System (HMIS). Ministry of Health and Family Welfare, Government of India. Accessed July 26, 2026. https://hmis.mohfw.gov.in/#!/standardReports
98. NHS Digital - Hospital episode Statistics (HES). Leeds: NHS England. Accessed July 26, 2026. https://digital.nhs.uk/data-and-information/data-tools-and-services/data-services/hospital-episode-statistics
99. Electronic Health Solutions (EHS) - Hakeem. Technology for better healthcare in Jordan. Accessed July 26, 2026. https://ehs.com.jo
100. Medical Data Vision - Nippon Life Insurance Company Group. Accessed July 26, 2026. https://en.mdv.co.jp
101. Public Health Scotland - Scottish Morbidity Record. Health intelligence and data management. Accessed July 26, 2026. https://publichealthscotland.scot
102. Research Data Assistance Center. Find, Request and Use CMS Data. Accessed July 26, 2026. https://resdac.org
103. Danish Health Data Authority. Better health through the use of data and digital coherence. Accessed July 26, 2026. https://sundhedsdatastyrelsen.dk
104. Department of Health. Statewide Planning and Research Cooperative System (SPARCS). Accessed July 26, 2026. https://www.health.ny.gov/statistics/sparcs/
105. International Federation for the Surgery of Obesity and Metabolic Disorders (IFSO). Accessed July 26, 2026. https://www.ifso.com
106. Ministry of Health - Oman. https://www.moh.gov. m
107. Rwanda Ministry of Health. https://www.moh.gov.rw

108. National Emergency Laparotomy Audit (NELA). https://www.nela.org.uk
