# Supplementary material for: Documenting lancet core surgical indicator on volumes: a scoping review of data collection methods
Source: BMJ Open. 2026 Nov 2;16(11):e118534. doi: 10.1136/bmjopen-2026-118534 (PMC13735403; doi:10.1136/bmjopen-2026-118534)
Supplement: Supplementary file 4 [file bmjopen-2026-118534.reviewer_comments.pdf]

PEER REVIEW HISTORY

BMJ Open publishes all reviews undertaken for accepted manuscripts. Reviewers are asked to complete a checklist review form and are provided with free text boxes to elaborate on their assessment. These free text comments are reproduced below.

ARTICLE DETAILS

Title (Provisional)

Documenting Lancet Core Surgical Indicator on Volumes: A Scoping Review of Data Collection Methods

Authors

Patil, Priti; van Duinen, Alex; Bakker, Juul; Nathani, Priyansh; Ng, Yuki J; Mohan, Monali; Bhushan, Pranav; Misra, Sidharth; Lindenbergh, Karel Cornelis; Bolkan , Håkon A; Roy, Nobhojit; Gadgil, Anita

---

VERSION 1 - REVIEW

**Reviewer**	**1**
**Name**	**Johnson, Eugenie**
**Affiliation**	**Newcastle University Institute for Health and Society, Newcastle University**
**Date**	**20-Mar-2026**
**COI**	**None**

Thank you to the authors for submitting this manuscript presenting a scoping review of reporting of surgical indicators. The review has strengths such as its detailed description of the study selection and data extraction process. However, I feel that the results could be explored in greater depth with further exploration of subgroups and that some of the statements within the discussion need further substantiation and clarification. I hope that my comments help improve the transparency and usefulness of the manuscript.

Major comments

1. Protocol: Please can the authors state whether they pre-planned the scoping review and if they published a protocol on a publicly-available platform, such as the OSF.

2. Search strategy; Limitations: Not including grey literature sources within the review could be a major limitation of the review. Given the topic area, I would expect that there would be a lot of information regarding surgical indicators in unpublished material that would not be found in the published literature. I appreciate that the authors have noted this as a

limitation of the review, but would appreciate greater explanation and reflection on the decision not to search grey literature sources given the nature of the topic.

3. Search strategy, dates: I note that articles were searched between 2014 and 2024. If the search was run in 2024, this means that the search is nearly two years out of date. What is the likelihood that new studies that could contribute to the conclusions of the scoping review have been published since 2024?

4. Methods, data synthesis: In line with recommendations from the JBI, please add a section at the end of the methods detailing how the extracted data was synthesised for presentation within the review.

5. Results: I believe that the results could be explored in greater detail. Currently, the results are presented for all 47 studies identified in total, but are there any other ways of analysing the data that would deepen the interpretation? For example, are there any differences in the level of reporting between different geographical regions, study settings or health systems being studied? I feel that a greater exploration and interrogation of the results would help to bring further nuance and interest to the article and allow the authors to discuss potential deficiencies in data collection more deeply.

6. Discussion: "Many studies have utilized the Utstein definition of surgery, which defines surgical procedures as those performed in operating theatres (OT) under anaesthesia". I appreciate the point the authors are making here about procedures not performed in theatre or under LA, but there is no mention of what definition of surgery the individual studies used within the results. Please either present these data and explore within the results, or remove this paragraph if no data can be provided to substantiate this.

7. Discussion: "There was considerable variation in how surgical procedures were classified across studies, with some relying on institutional coding systems and others using national or international classifications." This is not apparent from the results, where 40 (85%) of studies did not specify a surgery coding mechanism. If this relates to the type of procedures tracked (e.g. specific, elective and emergency), then greater nuance in the results is required to fully explore how surgeries were classified across studies.

8. Supplementary material 2: For this supplementary table, please can the authors provide further information about the characteristics of each of the studies. At a minimum, please add: the specific study design; setting (e.g. secondary or tertiary care); the period when data were collected in each study; the health setting; and health system. These details will help the reader to better understand the evidence base included within the review.

Minor comments

1. What is already known on this topic: There is a typo in the third line, "estimates is highly depends". Please can this be amended.

2. Introduction, general comment: It would be useful in the introduction to highlight the novelty of the scoping review. Have there been any previous reviews in this area and, if so,

how does this review add to the knowledge base and differentiate itself from these previous works?

3. Introduction, first line: While it is good to highlight the specific burden of inadequate surgical services in LMICs, since the study does not focus solely on surgical volume in LMICs, this sentence could be moved further down the paragraph or presented alongside a global statistic.

4. PRISMA-ScR: The authors state that they reported the review according to PRISMA-ScR guidelines but have provided a PRISMA 2020 checklist for systematic reviews of interventions. Please provide a completed PRISMA-ScR checklist.

5. Table 1, eligibility criteria: I have a couple of points regarding the eligibility criteria that I believe could be clarified further by the authors.

- What was the justification for only searching for studies between 2014 and 2024?

- It would be useful to know what types of surgical volume indicators were considered eligible for the review.

- "Qualitative, quantitative and modelling studies": please provide further information on the specific study designs included within the review (e.g. cohort studies, case control).

- "Studies based on primary as well as secondary data sources were included": It is not currently clear what is meant by this; please can this be explained further.

- "Single institution studies that describe data collection methods": By stating this, the implication that multicentre studies may have been excluded, though this is unlikely to be the case. This may need rephrasing to clarify that both single- and multicentre studies were eligible.

6. Discussion, first paragraph: "A systematic review reveals significant heterogeneity in the sources, methodologies used for collecting surgical volume data, and inconsistent reporting has impeded comparison between countries." The Patil 2023 review focuses specifically on LMICs and I think it is worth noting this here for added clarity.

7. Limitations: Critical appraisal is optional for scoping reviews as formal synthesis is not required, as noted by the JBI; this does not need to be mentioned as a limitation.

8. Limitations: Please reflect on the limitations of not involving patients and the public in the design, conduct or reporting of the scoping review.

**Reviewer**	**2**
**Name**	**Rausei, Stefano**
**Affiliation**	**ASST della Valle Olona**
**Date**	**20-Apr-2026**

**COI**	**None**

Patil and colleagues present an international study about the methodology of documentation of surgical volume in the world. The study is original and interesting. I have just some comments/suggestions:

1) Authors should update their literature research beyond 2024;

2) They should include some arguments as to why Europe and South America are contributing insufficiently to this issue and try to clarify the causes of such significant regional differences.

**Reviewer**	**3**
**Name**	**Oo, Aung Myint**
**Affiliation**	**Tan Tock Seng Hospital, General Surgery**
**Date**	**11-May-2026**
**COI**	**None**

Thanks for your scoping review. Your review demonstrated the urgent needs of standardization of definition and comprehensive data collections on surgical procedures especially in the developing countries where EMR/ EHR systems are not avaiable.

Suggest to include more information about the WHO and other global initiatives/strategies to address the accesible surgery and safe surgery will be great.

Thank you.

VERSION 1 - AUTHOR RESPONSE

Reviewer: 1 - Ms. Eugenie Johnson, Newcastle University Institute for Health and Society

Comments to the Author:

SN	Reviewer's comments	Authors' reply	Ref
	Thank you to the authors for submitting this manuscript presenting a scoping review of reporting of surgical indicators. The review has strengths such as its detailed description of the study selection and data extraction process. However, I feel that the results could be explored in greater depth with further		

SN	Reviewer's comments	Authors' reply	Ref
	exploration of subgroups and that some of the statements within the discussion need further substantiation and clarification. I hope that my comments help improve the transparency and usefulness of the manuscript.		
	**Major comments**		
1	Protocol: Please can the authors state whether they pre-planned the scoping review and if they published a protocol on a publicly-available platform, such as the OSF.	Thank you for this comment. Although the scoping review was pre-planned by the authors, a protocol was not published or registered on a publicly available platform such as the Open Science Framework (OSF).	
2	Search strategy; Limitations: Not including grey literature sources within the review could be a major limitation of the review. Given the topic area, I would expect that there would be a lot of information regarding surgical indicators in unpublished material that would not be found in the published literature. I appreciate that the authors have noted this as a limitation of the review, but would appreciate greater explanation and reflection on the decision not to search grey literature sources given the nature of the topic.	Thank you for pointing out this. We agree that, given the nature of the topic, grey literature is an important source of information on surgical volume data collection methodologies, particularly through government reports, policy documents, and organizational publications that may not be available in the peer-reviewed literature. In response to this comment, we expanded our search strategy to include relevant grey literature sources. This resulted in the inclusion of 23 grey literature documents, which have been incorporated into the review. Accordingly, the search strategy, eligibility criteria, Results, Discussion, and Strengths and Limitations sections have been revised to reflect the inclusion of grey literature. As a result, the previous limitation regarding the exclusion of grey literature has been removed from the manuscript.	Page 30
3	Search strategy, dates: I note that articles were searched between 2014 and 2024. If the search was	Thank you for this important observation. We agree that an updated search was necessary	Page 8

SN	Reviewer's comments	Authors' reply	Ref
	run in 2024, this means that the search is nearly two years out of date. What is the likelihood that new studies that could contribute to the conclusions of the scoping review have been published since 2024?	to ensure that the review reflects the most recent evidence. In response to this comment, we re-ran the search across all databases and grey literature sources, extending the search period from January 2014 to April 2026. Newly identified records were screened using the same eligibility criteria, and relevant studies and grey literature were included in the review. This update resulted in the inclusion of additional evidence, and the Methods, Results, Discussion, and supplementary materials have been revised accordingly to reflect the updated search.	
4	Methods, data synthesis: In line with recommendations from the JBI, please add a section at the end of the methods detailing how the extracted data was synthesised for presentation within the review.	Thank you for this suggestion. In accordance with the JBI guidance for scoping reviews, we have added a Data synthesis section at the end of the Methods. This section describes how the extracted data were synthesized and presented, including the use of descriptive summaries and thematic categorization of the identified methodologies.	Page 12
5	Results: I believe that the results could be explored in greater detail. Currently, the results are presented for all 47 studies identified in total, but are there any other ways of analysing the data that would deepen the interpretation? For example, are there any differences in the level of reporting between different geographical regions, study settings or health systems being studied? I feel that a greater exploration and interrogation of the results would help to bring further nuance and interest to the article and allow the authors to	Thank you for this valuable suggestion. As suggested, we have upgraded our search strategy to include the latest articles. Also, as mentioned in this comment, we have expanded the results section to provide a more detailed synthesis of the methodological characteristics of the included evidences. A new methodological characteristics table (Table 3, Supplementary table 2 and 3) has been added along with the revised results.	Page 13

SN	Reviewer's comments	Authors' reply	Ref
	discuss potential deficiencies in data collection more deeply.		
6	Discussion: "Many studies have utilized the Utstein definition of surgery, which defines surgical procedures as those performed in operating theatres (OT) under anaesthesia". I appreciate the point the authors are making here about procedures not performed in theatre or under LA, but there is no mention of what definition of surgery the individual studies used within the results. Please either present these data and explore within the results, or remove this paragraph if no data can be provided to substantiate this.	Thank you for this important observation. We agree that the Discussion should be supported by the review findings. Accordingly, we extracted and summarized the definitions of surgery reported by the included studies, and these data have now been presented in the Results (Table 3/Supplementary Table 2 and 3). The Discussion has been revised to interpret these findings and discuss the implications of the observed variation in the definitions of surgery and surgical volume.	Page 26
7	Discussion: "There was considerable variation in how surgical procedures were classified across studies, with some relying on institutional coding systems and others using national or international classifications." This is not apparent from the results, where 40 (85%) of studies did not specify a surgery coding mechanism. If this relates to the type of procedures tracked (e.g. specific, elective and emergency), then greater nuance in the results is required to fully explore how surgeries were classified across studies.	Thank you for this insightful comment. We agree that the original wording in the Discussion was not adequately supported by the Results. In response, we re-examined the extracted data and revised both the Results and Discussion. The Results now clearly distinguish between studies that explicitly reported the use of standardized coding systems and those that did not report any coding mechanism. The Discussion has been modified to reflect the review findings more accurately by emphasizing the limited reporting of surgical coding mechanisms rather than implying widespread variation in coding systems. We have also clarified the distinction between surgical coding systems and the classification of procedures where relevant.	Page 26
8	Supplementary material 2: For this supplementary table, please can the authors provide further information about the	Thank you for this helpful suggestion. We agree that additional study characteristics improve the interpretation of	Supplementary File-2

SN	Reviewer's comments	Authors' reply	Ref
	characteristics of each of the studies. At a minimum, please add: the specific study design; setting (e.g. secondary or tertiary care); the period when data were collected in each study; the health setting; and health system. These details will help the reader to better understand the evidence base included within the review.	the evidence base. Accordingly, we have revised Supplementary Material 2 to include the specific study design, period of data collection, health facility setting, and health system characteristics, where reported.	
	**Minor comments**		
1	What is already known on this topic: There is a typo in the third line, "estimates is highly depends". Please can this be amended.	We have removed this section as per the guidelines of the journal.	Page 4
2	Introduction, general comment: It would be useful in the introduction to highlight the novelty of the scoping review. Have there been any previous reviews in this area and, if so, how does this review add to the knowledge base and differentiate itself from these previous works?	Revised rationale has been updated into the introduction last two para.	Page 7
3	Introduction, first line: While it is good to highlight the specific burden of inadequate surgical services in LMICs, since the study does not focus solely on surgical volume in LMICs, this sentence could be moved further down the paragraph or presented alongside a global statistic.	Thank you for this helpful suggestion. We agree that the Introduction should first establish the global context before highlighting the disproportionate burden in LMICs. Accordingly, we have revised the opening paragraph to introduce the global burden of inadequate access to surgical care, followed by the LMIC-specific estimate of approximately 4.7 million avoidable deaths annually.	Page 7
4	PRISMA-ScR: The authors state that they reported the review according to PRISMA-ScR guidelines but have provided a PRISMA 2020 checklist for systematic reviews of interventions. Please provide a completed PRISMA-ScR checklist.	Apologies for this mistake. Updated PRISMA-ScR checklist included as suggested.	

SN	Reviewer's comments	Authors' reply	Ref
5	Table 1, eligibility criteria: I have a couple of points regarding the eligibility criteria that I believe could be clarified further by the authors.		
	What was the justification for only searching for studies between 2014 and 2024?	Thank you for pointing this out. We have now revised our search period to include articles up to April 2026.	Page 10
	It would be useful to know what types of surgical volume indicators were considered eligible for the review.	Point revised as suggested.	Page 10
	"Qualitative, quantitative and modelling studies": please provide further information on the specific study designs included within the review (e.g. cohort studies, case control).	Thank you for this suggestion. We have revised the eligibility criteria section to provide additional details regarding the specific study designs included in the review, such as cross-sectional, cohort, survey-based, registry-based, and modelling studies.	Page 10
	"Studies based on primary as well as secondary data sources were included": It is not currently clear what is meant by this; please can this be explained further.	Thank you for this comment. We have revised the eligibility criteria section to further clarify the meaning of primary and secondary data sources and have provided examples of the types of data sources included in the review.	Page 11
	"Single institution studies that describe data collection methods": By stating this, the implication that multicentre studies may have been excluded, though this is unlikely to be the case. This may need rephrasing to clarify that both single- and multicentre studies were eligible.	Thank you for this clarification. We have revised the wording to clearly indicate that both single-institution and multi-centre studies were eligible for inclusion in the review.	Page 11
6	Discussion, first paragraph: "A systematic review reveals significant heterogeneity in the sources, methodologies used for collecting surgical volume data, and inconsistent reporting has impeded comparison between countries." The Patil 2023 review	Thank you for this helpful suggestion. We have revised the discussion section to clarify that the Patil et al. 2023 review focused specifically on low- and middle-income countries (LMICs).	Page 21

SN	Reviewer's comments	Authors' reply	Ref
	focuses specifically on LMICs and I think it is worth noting this here for added clarity.		
7	Limitations: Critical appraisal is optional for scoping reviews as formal synthesis is not required, as noted by the JBI; this does not need to be mentioned as a limitation.	Thank you for this clarification. We agree that critical appraisal is optional in scoping reviews according to JBI guidance. Accordingly, this point has been removed from the limitations section.	Page 30
8	Limitations: Please reflect on the limitations of not involving patients and the public in the design, conduct or reporting of the scoping review.	Thank you for this suggestion. We have revised the Strengths and Limitations section to acknowledge that patients and members of the public were not involved in the design, conduct, or reporting of this scoping review. We have also reflected on the potential implications of the absence of stakeholder involvement and noted that future methodological research may benefit from engaging relevant	Page 30

Reviewer: 2 - Dr. Stefano Rausei, ASST della Valle Olona

Comments to the Author:

SN	Reviewer's comments	Authors' reply	Ref
	Patil and colleagues present an international study about the methodology of documentation of surgical volume in the world. The study is original and interesting. I have just some comments/suggestions:		
1	Authors should update their literature research beyond 2024;	Thank you for this suggestion. We have updated the literature search to include studies published up to April 2026 and also included relevant grey literature as suggested in another comment. Additional eligible evidence sources were identified and incorporated into the review. The Methods, Results, tables, and Supplementary Materials have	Page 8

SN	Reviewer’s comments	Authors’ reply	Ref
		been revised accordingly to reflect the updated output.	
2	They should include some arguments as to why Europe and South America are contributing insufficiently to this issue and try to clarify the causes of such significant regional differences.	Thank you for this valuable comment. We have updated our search by including recently published studies and relevant grey literature. As a result, the representation from these regions has improved. The revised review now includes 2 original studies and 6 grey literature sources from Europe, and 2 original studies and 1 grey literature source from South America.<br>We also considered discussing the possible reasons for the lower number of studies from these regions. However, we felt that any explanation would be speculative, as our review was not designed to investigate the reasons for regional differences. The number of included studies may reflect publication practices, availability of methodological reports, or the use of well-established health information systems where the data collection methods are not published as separate methodological papers. Since we cannot confirm these reasons based on the available evidence, we have not added them to the manuscript.	

Reviewer: 3 - Dr. Aung Myint Oo, Tan Tock Seng Hospital

Comments to the Author:

SN	Reviewer’s comments	Authors’ reply	Ref
	Thanks for your scoping review. Your review demonstrated the urgent needs of standardization of definition and comprehensive data collections on surgical procedures especially in the developing countries where EMR/ EHR systems are not	Thank you for your positive comments. We agree that placing our findings within the context of global surgical initiatives strengthens the manuscript. Accordingly, we have revised the Discussion to	Page 31

	available.<br>Suggest to include more information about the WHO and other global initiatives/strategies to address the accessible surgery and safe surgery will be great.<br>Thank you.	include information on relevant WHO and global surgery initiatives, including the WHO Resolution WHA68.15 on strengthening emergency and essential surgical care and anaesthesia, the Lancet Commission on Global Surgery (LCoGS), and the WHO Action Framework for Safe and Affordable Surgery. We have highlighted how standardized definitions, robust surgical information systems, and comprehensive data collection are fundamental to monitoring surgical system performance and supporting these global efforts to improve access to safe surgical care.	

VERSION 2 - REVIEW

Reviewer 1

Name Johnson, Eugenie

Affiliation Newcastle University Institute for Health and Society, Newcastle University

Date 12-Aug-2026

COI

Many thanks to the authors for their careful consideration of the previous round of review comment, including incorporating major updates to the search strategies and eligibility criteria. This was a very large amount of work and I believe it has greatly enhanced the manuscript as a whole. My latest comments are more minor and mainly focus on clarifying specific points within the methods and results. I hope that these comments will prove to be useful in this regard.

Major comments

1. Reporting of results: Thank you to the authors for greatly expanding upon their original results and reflecting on one of my previous comments regarding the depth of the discussion; I believe the manuscript is greatly enhanced because of these changes. However, there is a lack of referencing throughout the results section. While I appreciate that this is a

large review with 83 studies, for transparency for the reader, it is recommended for references of included studies be added throughout the results wherever percentages of numbers of included studies are mentioned. Please can the authors consider adding these in to enhance the overall transparency of the review.

Minor comments

1. Strengths and limitations of the review: In the newly-added bullet points after the abstract, there is currently only one limitation mentioned, despite there being three different limitations reported in the Discussion section. Given that the Editor has noted that it is possible to include up to 5 bullet points in this section, please can the authors use the final bullet point to detail other limitations of the review.

2. Methods, eligibility criteria: Thank you to the authors for considering my previous comments on the eligibility criteria of the review. Please can the authors further clarify why they chose a cut-off date of January 2014 as part of the eligibility criteria? Could relevant information from before this time have been missed due to this criterion?

3. Methods, data synthesis: Thank you to the authors for adding the new section regarding data synthesis. Having read the results, it would be useful for the authors to mention that they conducted a subgroup analysis of LIC/LMICs with UMICs/HICs and the rationale behind comparing these, for additional transparency in the methods.

4. Results, second paragraph: It is not entirely clear what is meant by the term “resources” in this section (for example, “multi-country resources”). Can this term be further clarified?

5. Results, Table 2: Thank you for updating this table, it is very helpful as an overview of the evidence base. In the grey literature column, it is surprising to me that there were no details in the grey literature identified about either the study setting or design. Can the authors clarify if, when it is started “NA”, are there places where this should be “NR” (not reported)? This should also be considered for the tables in supplementary materials, which also use “NA”.

6. Results: “Emergency, elective, and essential surgery indicators were comparatively uncommon.” Can the authors please provide percentages for these surgery indicators to demonstrate how uncommon the reporting of these were? In addition, would this information be better placed in the following paragraph, where specific types and definitions of surgery are discussed in further depth?

7. Structure of results: It may be useful to include further subheadings within the results to clearly demarcate new topics (for example, where the comparison between LIC and LMICs with UMICs and HICs is introduced).

8. Discussion, main findings: “without consistently describing what was included in the numerator.” In the results, it was stated that “several resources reported total numbers of procedures with or without population denominators”; numerators are not mentioned

elsewhere in the manuscript. Please can this discrepancy be clarified or amended appropriately.

9. Discussion, main findings: “This resulted in underestimation of the true surgical workload and limiting the ability of surgical volume indicators to reflect the overall burden of surgical care.” Given that the purpose of the scoping review was to assess the overall reporting of surgeries, rather than the actual surgical workload, it is unclear that this is possible to conclude from the data presented. It may be possible to suggest that using the Utstein classification “could” lead to an underestimation of workload. Please can the authors revise appropriately.

10. Strengths and limitations of the review: The authors have noted in their response to peer-review comments that a protocol was not pre-published in a publicly-available platform. It may be worth adding to the limitations that, while planned internally, the review protocol was not openly published.

11. Supplementary material 2: Can the authors please clarify the difference between the Utstein definition of surgery and “Surgeries requiring general, regional, spinal, or local anaesthesia and performed in an operating theatre” in this supplementary table?

12. Supplementary material 2: When discussing the ‘Common limitations’, are these as defined by the authors of the included studies and reports, or defined by the review authors? This may also be useful to clarify within the methods of the main manuscript, in the data extraction section.

13. General comment: There are a few typos throughout the document. I appreciate that the manuscript has undergone a major update and that some typos would be likely as a result, though a quick proofread to ensure correct spelling would be helpful.

VERSION 2 - AUTHOR RESPONSE

Reviewer: 1 - Ms. Eugenie Johnson, Newcastle University Institute for Health and Society

Comments to the Author:

SN	Reviewer’s comments	Authors’ reply	Ref
	Many thanks to the authors for their careful consideration of the previous round of review comment, including incorporating major updates to the search strategies and eligibility criteria. This was a very large amount of work and I believe it has greatly enhanced the manuscript as a whole. My latest comments are more minor and mainly focus on	We sincerely thank the reviewer for the positive and encouraging feedback. We appreciate your recognition of the substantial revisions made to the manuscript. Your constructive comments have helped us further improve the clarity and quality of the review. Our point-by-point responses to the	

SN	Reviewer's comments	Authors' reply	Ref
	clarifying specific points within the methods and results. I hope that these comments will prove to be useful in this regard.	specific comments are provided below.	
	Major comments		
1	Reporting of results: Thank you to the authors for greatly expanding upon their original results and reflecting on one of my previous comments regarding the depth of the discussion; I believe the manuscript is greatly enhanced because of these changes. However, there is a lack of referencing throughout the results section. While I appreciate that this is a large review with 83 studies, for transparency for the reader, it is recommended for references of included studies be added throughout the results wherever percentages of numbers of included studies are mentioned. Please can the authors consider adding these in to enhance the overall transparency of the review.	Thank you for highlighting the importance of traceability in the Results. We agree that readers should be able to identify the evidence underlying each descriptive finding. In this review, however, most percentages and frequencies are aggregate results derived from the structured extraction and synthesis of a large number of the 83 included sources, rather than findings attributable to a single study or a small number of individual studies. Adding the full set of contributing citations after each numerical result would therefore result in repeated and extensive citation clusters throughout the Results, without necessarily improving the reader's ability to interpret the synthesis.<br><br>We have therefore taken a source-level traceability approach. The main text presents the aggregate descriptive findings, while the relevant supplementary tables provide the study-level characteristics and methodological data from which these summaries were derived. To make this link clearer for readers, we have strengthened cross-referencing from the Results to the relevant supplementary tables. Individual references are retained where a statement relates to a specific study, definition, methodological approach, or a	No Action

SN	Reviewer's comments	Authors' reply	Ref
		small identifiable group of sources.<br><br>We hope this approach provides transparency and reproducibility while keeping the main Results sufficiently readable.	
	Minor comments		
1	Strengths and limitations of the review: In the newly-added bullet points after the abstract, there is currently only one limitation mentioned, despite there being three different limitations reported in the Discussion section. Given that the Editor has noted that it is possible to include up to 5 bullet points in this section, please can the authors use the final bullet point to detail other limitations of the review.	Thank you for this suggestion. We have now added an additional limitation to the Strengths and limitations section to better reflect the limitations discussed in the manuscript.	Page 5 - Line 16
2	Methods, eligibility criteria: Thank you to the authors for considering my previous comments on the eligibility criteria of the review. Please can the authors further clarify why they chose a cut-off date of January 2014 as part of the eligibility criteria? Could relevant information from before this time have been missed due to this criterion?	Thank you for this comment. We selected January 2014 as the start date to focus the review on contemporary methods (current approaches) of surgical volume data collection. Over the past decade, there have been substantial advances in electronic medical records, health information systems, surgical coding practices, and digital data collection. We therefore anticipated that methodologies reported during this period would be more relevant to current surgical data collection practices. While we acknowledge that some earlier studies may have been excluded, we considered this time restriction appropriate to ensure that the review reflects current approaches and remains focused and relevant. We have clarified this rationale in the Methods section.	No action
3	Methods, data synthesis: Thank you to the authors for adding the new section regarding data synthesis.	Thank you for this suggestion. We agree that this comparison should be described more	Page 12 - Line 5

SN	Reviewer's comments	Authors' reply	Ref
	Having read the results, it would be useful for the authors to mention that they conducted a subgroup analysis of LIC/LMICs with UMICs/HICs and the rationale behind comparing these, for additional transparency in the methods.	clearly in the Methods. We have revised the Data Synthesis section to state that a descriptive comparison was undertaken between studies conducted in LICs/LMICs and those conducted in UMICs/HICs.	
4	Results, second paragraph: It is not entirely clear what is meant by the term "resources" in this section (for example, "multi-country resources"). Can this term be further clarified?	Thank you for pointing this out. We agree that the term "resources" was ambiguous. We have replaced it with "grey literature sources" and clarified that these refer to multi-country grey literature documents and web-based sources reporting surgical volume data from countries across different World Bank income groups.	Page 13 - Line 3
5	Results, Table 2: Thank you for updating this table, it is very helpful as an overview of the evidence base. In the grey literature column, it is surprising to me that there were no details in the grey literature identified about either the study setting or design. Can the authors clarify if, when it is started "NA", are there places where this should be "NR" (not reported)? This should also be considered for the tables in supplementary materials, which also use "NA".	Thank you for this helpful observation. We agree that "Not Applicable (NA)" and "Not Reported (NR)" represent different situations. We have reviewed Table 2 and the supplementary tables and revised the terminology accordingly. Entries have been changed to NR where the information was not reported or available in the source. We have also added the full forms of both abbreviations in the table footnotes for clarity.	Page 13 - Line 10
6	Results: "Emergency, elective, and essential surgery indicators were comparatively uncommon." Can the authors please provide percentages for these surgery indicators to demonstrate how uncommon the reporting of these were? In addition, would this information be better placed in the following paragraph, where specific types and definitions of surgery are discussed in further depth?	Thank you for this helpful suggestion. We have revised the sentence to clarify that 10.8% of the included studies reported surgical volume according to either essential, emergency, or elective surgery categories. We have also moved this statement to the subsequent paragraph, where the classification and reporting of surgical procedures are described.	Page 14 - Line 15
7	Structure of results: It may be useful to include further subheadings within the results to clearly	Thank you for this helpful suggestion. We agree that additional subheadings improve	Throughout results section

SN	Reviewer's comments	Authors' reply	Ref
	demarcate new topics (for example, where the comparison between LIC and LMICs with UMICs and HICs is introduced).	the readability and organisation of the Results section. Accordingly, we have introduced appropriate subheadings, including a separate subheading for the comparison of studies conducted in LICs/LMICs and UMICs/HICs, to better distinguish the different topics presented.	
8	Discussion, main findings: "without consistently describing what was included in the numerator." In the results, it was stated that "several resources reported total numbers of procedures with or without population denominators"; numerators are not mentioned elsewhere in the manuscript. Please can this discrepancy be clarified or amended appropriately.	Thank you for this observation. We agree that the term "numerator" had not been explicitly introduced in the Results and could be unclear. We have therefore revised the Discussion to replace this terminology with "which surgical procedures were included in the reported surgical volume estimates".	Page 18 - Line 18
9	Discussion, main findings: "This resulted in underestimation of the true surgical workload and limiting the ability of surgical volume indicators to reflect the overall burden of surgical care." Given that the purpose of the scoping review was to assess the overall reporting of surgeries, rather than the actual surgical workload, it is unclear that this is possible to conclude from the data presented. It may be possible to suggest that using the Utstein classification "could" lead to an underestimation of workload. Please can the authors revise appropriately.	Thank you for this observation. We agree that our review was designed to examine methods used to collect and report surgical volume rather than to quantify the true surgical workload. Accordingly, we have revised the Discussion to avoid implying a demonstrated effect. The statement now indicates that use of the Utstein definition could lead to an underestimation of the overall surgical workload and may limit the ability of surgical volume indicators to fully reflect the burden of surgical care.	Page 20 - Line 6
10	Strengths and limitations of the review: The authors have noted in their response to peer-review comments that a protocol was not pre-published in a publicly-available platform. It may be worth adding to the limitations that, while planned internally, the review protocol was not openly published.	Thank you for this valuable suggestion. We agree that public availability of a review protocol enhances transparency. Although a protocol was developed prior to conducting the review, it was not published on a publicly accessible platform. We have now	Page 24 - Line 1

SN	Reviewer's comments	Authors' reply	Ref
		acknowledged this as a limitation in the manuscript.	
11	Supplementary material 2: Can the authors please clarify the difference between the Utstein definition of surgery and "Surgeries requiring general, regional, spinal, or local anaesthesia and performed in an operating theatre" in this supplementary table?	Thank you for identifying this important point. We have now modified this section to combine both the surgery definitions as a Utstein definition and revised the results accordingly.	Page 11 - Line 15
12	Supplementary material 2: When discussing the 'Common limitations', are these as defined by the authors of the included studies and reports, or defined by the review authors? This may also be useful to clarify within the methods of the main manuscript, in the data extraction section.	Thank you for this comment. The "Common limitations" presented in Supplementary Table 2 represent a combination of limitations explicitly reported by the authors of the included studies and grey literature sources, as well as additional methodological limitations identified by the review authors based on the reported methods and characteristics of each source. We agree that this required clarification and have revised the Data Extraction section of the Methods to describe how these limitations were identified and summarised.	Page 16 - Line 5
13	General comment: There are a few typos throughout the document. I appreciate that the manuscript has undergone a major update and that some typos would be likely as a result, though a quick proofread to ensure correct spelling would be helpful.	I would greatly appreciate it if someone from the team could kindly go through the manuscript once more, specifically to check for any grammar, spelling, or typographical errors. Given the extensive revisions, a fresh pair of eyes would be extremely helpful before the final decision.	Throughout the manuscript
