# Supplementary material for: Documenting lancet core surgical indicator on volumes: a scoping review of data collection methods
Source: BMJ Open. 2026 Nov 2;16(11):e118534. doi: 10.1136/bmjopen-2026-118534 (PMC13735403; doi:10.1136/bmjopen-2026-118534)
Supplement: Supplementary file 5 [file bmjopen-16-11-s001.docx]

**Documenting Lancet Core Surgical Indicator on Volumes: A Scoping Review of Data Collection Methods**

**Supplementary Table 1: Article Search Criteria**

**PubMed:**

| Search number | Query | Results |
| --- | --- | --- |
| 9 | (((#1 AND #8) NOT #3) AND (("2014/01/01"[Date - Publication] : "3000"[Date - Publication]))) AND (English[Language]) | 2,377 |
| 8 | (volume[Title]) OR (rate*[Title]) OR (Enumerat*[Title]) OR (measur*[Title]) OR (modelling[Title]) OR (metrics[Title]) OR ("unmet need*"[Title]) OR ("total number*"[Title]) | 7,53,151 |
| 3 | (*cardiac*[Title]) OR (adrenal[Title]) OR (aest*[Title]) OR (aneu*[Title]) OR (angio*[Title]) OR (ankl*[Title]) OR (anti*[Title]) OR (Aortic[Title]) OR (aqua*[Title]) OR (arte*[Title]) OR (arth*[Title]) OR (arthro*[Title]) OR (atria*[Title]) OR (augm*[Title]) OR (bari*[Title]) OR (bloo*[Title]) OR (brac*[Title]) OR (breast*[Title]) OR (calc*[Title]) OR (cancer*[Title]) OR (carci*[Title]) OR (cardio*[Title]) OR (cata*[Title]) OR (cerebro*[Title]) OR (cerv*[Title]) OR (chem*[Title]) OR (chole*[Title]) OR (chro*[Title]) OR (cirr*[Title]) OR (clef*[Title]) OR (cont*[Title]) OR (corn*[Title]) OR (coro*[Title]) OR (crani*[Title]) OR (cryst*[Title]) OR (cyst*[Title]) OR (electro*[Title]) OR (emph*[Title]) OR (endo*[Title]) OR (exci*[Title]) OR (eye[Title]) OR (femo*[Title]) OR (fist*[Title]) OR (foot*[Title]) OR (gall*[Title]) OR (Gastric[Title]) OR (glau*[Title]) OR (glom*[Title]) OR (head*[Title]) OR (hear*[Title]) OR (heart*[Title]) OR (hip[Title]) OR (hipp*[Title]) OR (hyper*[Title]) OR (hypo*[Title]) OR (hyst*[Title]) OR (infection*[Title]) OR (inte*[Title]) OR (intra*[Title]) OR (join*[Title]) OR (kidn*[Title]) OR (lase*[Title]) OR (lipo*[Title]) OR (live*[Title]) OR (lump*[Title]) OR (lung*[Title]) OR (lymp*[Title]) OR (macro*[Title]) OR (marg*[Title]) OR (mech*[Title]) OR (meta*[Title]) OR (metas*[Title]) OR (micro*[Title]) OR (mitral*[Title]) OR (muta*[Title]) OR (neck*[Title]) OR (neph*[Title]) OR (nerv*[Title]) OR (neur*[Title]) OR (nutr*[Title]) OR (obes*[Title]) OR (onco*[Title]) OR (opht*[Title]) OR (orth*[Title]) OR (pancr*[Title]) OR (para*[Title]) OR (path*[Title]) OR (peni*[Title]) OR (peri*[Title]) OR (phaco*[Title]) OR (phar*[Title]) OR (pitu*[Title]) OR (plast*[Title]) OR (plat*[Title]) OR (post*[Title]) OR (prost*[Title]) OR (pulm*[Title]) OR (pump*[Title]) OR (radio*[Title]) OR (recon*[Title]) OR (rect*[Title]) OR (renal*[Title]) OR (reti*[Title]) OR (rib[Title]) OR (robo*[Title]) OR (sarcoma[Title]) OR (schw*[Title]) OR (spine*[Title]) OR (ster*[Title]) OR (stone[Title]) OR (stroke*[Title]) OR (testi*[Title]) OR (thor*[Title]) OR (throm*[Title]) OR (thyro*[Title]) OR (tibi*[Title]) OR (Ulce*[Title]) OR (uret*[Title]) OR (urol*[Title]) OR (vasc*[Title]) OR (venti*[Title]) OR (Tumor[Title]) OR (Suicide[Title]) OR (Vaccin*[Title]) OR (Malaria[Title]) OR (Mental health[Title]) OR (clinical trial*[Title]) OR (telemedicine[Title]) OR (abortion[Title]) OR (reproductive[Title]) OR (stillbirth[Title]) | 2,09,49,449 |
| 1 | (Surgical[Title]) OR (procedure*[Title]) OR (surger*[Title]) OR (operation*[Title]) | 9,02,132 |

**Scopus**

( ( TITLE ( surgical ) OR TITLE ( procedure* ) OR TITLE ( surger* ) OR TITLE ( operation* ) )

AND

( TITLE ( volume ) OR TITLE ( rate* ) OR TITLE ( enumerat* ) OR TITLE ( measur* ) OR TITLE ( modelling ) OR TITLE ( "total number*" ) OR TITLE ( metrics ) OR TITLE ( "unmet need*" ) ) )

AND NOT

( TITLE ( *cardiac* ) OR TITLE ( adrenal ) OR TITLE ( aest* ) OR TITLE ( aneu* ) OR TITLE ( angio* ) OR TITLE ( ankl* ) OR TITLE ( anti* ) OR TITLE ( aortic ) OR TITLE ( aqua* ) OR TITLE ( arte* ) OR TITLE ( arth* ) OR TITLE ( arthro* ) OR TITLE ( atria* ) OR TITLE ( augm* ) OR TITLE ( bari* ) OR TITLE ( bloo* ) OR TITLE ( brac* ) OR TITLE ( breast* ) OR TITLE ( calc* ) OR TITLE ( cancer* ) OR TITLE ( carci* ) OR TITLE ( cardio* ) OR TITLE ( cata* ) OR TITLE ( cerebro* ) OR TITLE ( cerv* ) OR TITLE ( chem* ) OR TITLE ( chole* ) OR TITLE ( chro* ) OR TITLE ( cirr* ) OR TITLE ( clef* ) OR TITLE ( cont* ) OR TITLE ( corn* ) OR TITLE ( coro* ) OR TITLE ( crani* ) OR TITLE ( cryst* ) OR TITLE ( cyst* ) OR TITLE ( electro* ) OR TITLE ( emph* ) OR TITLE ( endo* ) OR TITLE ( exci* ) OR TITLE ( eye ) OR TITLE ( femo* ) OR TITLE ( fist* ) OR TITLE ( foot* ) OR TITLE ( gall* ) OR TITLE ( gastric ) OR TITLE ( glau* ) OR TITLE ( glom* ) OR TITLE ( head* ) OR TITLE ( hear* ) OR TITLE ( heart* ) OR TITLE ( hip ) OR TITLE ( hipp* ) OR TITLE ( hyper* ) OR TITLE ( hypo* ) OR TITLE ( hyst* ) OR TITLE ( infection* ) OR TITLE ( inte* ) OR TITLE ( intra* ) OR TITLE ( join* ) OR TITLE ( kidn* ) OR TITLE ( lase* ) OR TITLE ( lipo* ) OR TITLE ( live* ) OR TITLE ( lump* ) OR TITLE ( lung* ) OR TITLE ( lymp* ) OR TITLE ( macro* ) OR TITLE ( marg* ) OR TITLE ( mech* ) OR TITLE ( meta* ) OR TITLE ( metas* ) OR TITLE ( micro* ) OR TITLE ( mitral* ) OR TITLE ( muta* ) OR TITLE ( neck* ) OR TITLE ( neph* ) OR TITLE ( nerv* ) OR TITLE ( neur* ) OR TITLE ( nutr* ) OR TITLE ( obes* ) OR TITLE ( onco* ) OR TITLE ( opht* ) OR TITLE ( orth* ) OR TITLE ( pancr* ) OR TITLE ( para* ) OR TITLE ( path* ) OR TITLE ( peni* ) OR TITLE ( peri* ) OR TITLE ( phaco* ) OR TITLE ( phar* ) OR TITLE ( pitu* ) OR TITLE ( plast* ) OR TITLE ( plat* ) OR TITLE ( post* ) OR TITLE ( prost* ) OR TITLE ( pulm* ) OR TITLE ( pump* ) OR TITLE ( radio* ) OR TITLE ( recon* ) OR TITLE ( rect* ) OR TITLE ( renal* ) OR TITLE ( reti* ) OR TITLE ( rib ) OR TITLE ( robo* ) OR TITLE ( sarcoma ) OR TITLE ( schw* ) OR TITLE ( spine* ) OR TITLE ( ster* ) OR TITLE ( stone ) OR TITLE ( stroke* ) OR TITLE ( testi* ) OR TITLE ( thor* ) OR TITLE ( throm* ) OR TITLE ( thyro* ) OR TITLE ( tibi* ) OR TITLE ( ulce* ) OR TITLE ( uret* ) OR TITLE ( urol* ) OR TITLE ( vasc* ) OR TITLE ( venti* ) OR TITLE ( tumor ) OR TITLE ( suicide ) OR TITLE ( vaccin* ) OR TITLE ( malaria ) OR TITLE ( mental AND health ) OR TITLE ( clinical AND trial* ) OR TITLE ( telemedicine ) OR TITLE ( abortion ) OR TITLE ( reproductive ) OR TITLE ( stillbirth ) )

AND

( LIMIT-TO ( SUBJAREA , "MEDI" ) OR LIMIT-TO ( SUBJAREA , "SOCI" ) OR LIMIT-TO ( SUBJAREA , "HEAL" ) OR LIMIT-TO ( SUBJAREA , "ECON" ) )

AND

( LIMIT-TO ( LANGUAGE , "English" ) ) AND ( LIMIT-TO ( OA , "all" ) )

**Web of Science**

((TI= (Surgical) OR TI= (procedure*) OR TI= (surger*) OR TI= (operation*))

AND

(TI= (volume) OR TI= (rate*) OR TI= (Enumerat*) OR TI= (measur*) OR TI= (modelling) OR TI= ("total number*") OR TI= (metrics) OR TI= ("unmet need*")))

NOT

(TI= (*cardiac*) OR TI= (adrenal) OR TI= (aest*) OR TI= (aneu*) OR TI= (angio*) OR TI= (ankl*) OR TI= (anti*) OR TI= (Aortic) OR TI= (aqua*) OR TI= (arte*) OR TI= (arth*) OR TI= (arthro*) OR TI= (atria*) OR TI= (augm*) OR TI= (bari*) OR TI= (bloo*) OR TI= (brac*) OR TI= (breast*) OR TI= (calc*) OR TI= (cancer*) OR TI= (carci*) OR TI= (cardio*) OR TI= (cata*) OR TI= (cerebro*) OR TI= (cerv*) OR TI= (chem*) OR TI= (chole*) OR TI= (chro*) OR TI= (cirr*) OR TI= (clef*) OR TI= (cont*) OR TI= (corn*) OR TI= (coro*) OR TI= (crani*) OR TI= (cryst*) OR TI= (cyst*) OR TI= (electro*) OR TI= (emph*) OR TI= (endo*) OR TI= (exci*) OR TI= (eye) OR TI= (femo*) OR TI= (fist*) OR TI= (foot*) OR TI= (gall*) OR TI= (Gastric) OR TI= (glau*) OR TI= (glom*) OR TI= (head*) OR TI= (hear*) OR TI= (heart*) OR TI= (hip) OR TI= (hipp*) OR TI= (hyper*) OR TI= (hypo*) OR TI= (hyst*) OR TI= (infection*) OR TI= (inte*) OR TI= (intra*) OR TI= (join*) OR TI= (kidn*) OR TI= (lase*) OR TI= (lipo*) OR TI= (live*) OR TI= (lump*) OR TI= (lung*) OR TI= (lymp*) OR TI= (macro*) OR TI= (marg*) OR TI= (mech*) OR TI= (meta*) OR TI= (metas*) OR TI= (micro*) OR TI= (mitral*) OR TI= (muta*) OR TI= (neck*) OR TI= (neph*) OR TI= (nerv*) OR TI= (neur*) OR TI= (nutr*) OR TI= (obes*) OR TI= (onco*) OR TI= (opht*) OR TI= (orth*) OR TI= (pancr*) OR TI= (para*) OR TI= (path*) OR TI= (peni*) OR TI= (peri*) OR TI= (phaco*) OR TI= (phar*) OR TI= (pitu*) OR TI= (plast*) OR TI= (plat*) OR TI= (post*) OR TI= (prost*) OR TI= (pulm*) OR TI= (pump*) OR TI= (radio*) OR TI= (recon*) OR TI= (rect*) OR TI= (renal*) OR TI= (reti*) OR TI= (rib) OR TI= (robo*) OR TI= (sarcoma) OR TI= (schw*) OR TI= (spine*) OR TI= (ster*) OR TI= (stone) OR TI= (stroke*) OR TI= (testi*) OR TI= (thor*) OR TI= (throm*) OR TI= (thyro*) OR TI= (tibi*) OR TI= (Ulce*) OR TI= (uret*) OR TI= (urol*) OR TI= (vasc*) OR TI= (venti*) OR TI= (Tumor) OR TI= (Suicide) OR TI= (Vaccin*) OR TI= (Malaria) OR TI= (Mental health) OR TI= (clinical trial*) OR TI= (telemedicine) OR TI= (abortion) OR TI= (reproductive) OR TI= (stillbirth) )

**Embase**

| No. | Query | Output | |
| --- | --- | --- | --- |
| 1 | 'surgical':ti OR 'procedure*':ti OR 'surger*':ti OR 'operation*':ti | 11,42,305 | |
| 2 | **'volume'**:ti OR **'rate*'**:ti OR **'enumerat*'**:ti OR **'measur*'**:ti OR **'modelling'**:ti OR **'metrics'**:ti OR **'unmet need*'**:ti OR **'total number*'**:ti | 9,41,655 | |
| 3 | cardiac*':ti OR 'adrenal':ti OR 'aest*':ti OR 'aneu*':ti OR 'angio*':ti OR 'ankl*':ti OR 'anti*':ti OR 'aortic':ti OR 'aqua*':ti OR 'arte*':ti OR 'arth*':ti OR 'arthro*':ti OR 'atria*':ti OR 'augm*':ti OR 'bari*':ti OR 'bloo*':ti OR 'brac*':ti OR 'breast*':ti OR 'calc*':ti OR 'cancer*':ti OR 'carci*':ti OR 'cardio*':ti OR 'cata*':ti OR 'cerebro*':ti OR 'cerv*':ti OR 'chem*':ti OR 'chole*':ti OR 'chro*':ti OR 'cirr*':ti OR 'clef*':ti OR 'cont*':ti OR 'corn*':ti OR 'coro*':ti OR 'crani*':ti OR 'cryst*':ti OR 'cyst*':ti OR 'electro*':ti OR 'emph*':ti OR 'endo*':ti OR 'exci*':ti OR 'eye':ti OR 'femo*':ti OR 'fist*':ti OR 'foot*':ti OR 'gall*':ti OR 'gastric':ti OR 'glau*':ti OR 'glom*':ti OR 'head*':ti OR 'hear*':ti OR 'heart*':ti OR 'hip':ti OR 'hipp*':ti OR 'hyper*':ti OR 'hypo*':ti OR 'hyst*':ti OR 'infection*':ti OR 'inte*':ti OR 'intra*':ti OR 'join*':ti OR 'kidn*':ti OR 'lase*':ti OR 'lipo*':ti OR 'live*':ti OR 'lump*':ti OR 'lung*':ti OR 'lymp*':ti OR 'macro*':ti OR 'marg*':ti OR 'mech*':ti OR 'meta*':ti OR 'metas*':ti OR 'micro*':ti OR 'mitral*':ti OR 'muta*':ti OR 'neck*':ti OR 'neph*':ti OR 'nerv*':ti OR 'neur*':ti OR 'nutr*':ti OR 'obes*':ti OR 'onco*':ti OR 'opht*':ti OR 'orth*':ti OR 'pancr*':ti OR 'para*':ti OR 'path*':ti OR 'peni*':ti OR 'peri*':ti OR 'phaco*':ti OR 'phar*':ti OR 'pitu*':ti OR 'plast*':ti OR 'plat*':ti OR 'post*':ti OR 'prost*':ti OR 'pulm*':ti OR 'pump*':ti OR 'radio*':ti OR 'recon*':ti OR 'rect*':ti OR 'renal*':ti OR 'reti*':ti OR 'rib':ti OR 'robo*':ti OR 'sarcoma':ti OR 'schw*':ti OR 'spine*':ti OR 'ster*':ti OR 'stone':ti OR 'stroke*':ti OR 'testi*':ti OR 'thor*':ti OR 'throm*':ti OR 'thyro*':ti OR 'tibi*':ti OR 'ulce*':ti OR 'uret*':ti OR 'urol*':ti OR 'vasc*':ti OR 'venti*':ti OR 'tumor':ti OR 'suicide':ti OR 'vaccin*':ti OR 'malaria':ti OR 'mental health':ti OR 'clinical trial*':ti OR 'telemedicine':ti OR 'abortion':ti OR 'reproductive':ti OR 'stillbirth':ti | 2,60,64,289 | |
| 4 | 1 AND 2 | 27,739 |  |
| 5 | 1 AND 2 NOT 3 | 6,684 |  |
| 6 | #1 AND #2 NOT #3 AND [2014-2024]/py | 3,367 |  |
| 7 | #1 AND #2 NOT #3 AND [2014-2024]/py AND [english]/lim | 3,307 |  |

**Cochrane**

| No. | Query | Output |
| --- | --- | --- |
| 1 | (Surgical) OR (procedure*) OR (surger*) OR (operation*) | 1,03,711 |
| 2 | (volume) OR (rate*) OR (Enumerat*) OR (measur*) OR (modelling) OR (metrics) OR (unmet NEXT need*) OR (total NEXT number*) | 67,247 |
| 3 | (cardiac*) OR (adrenal) OR (aest*) OR (aneu*) OR (angio*) OR (ankl*) OR (anti*) OR (Aortic) OR (aqua*) OR (arte*) OR (arth*) OR (arthro*) OR (atria*) OR (augm*) OR (bari*) OR (bloo*) OR (brac*) OR (breast*) OR (calc*) OR (cancer*) OR (carci*) OR (cardio*) OR (cata*) OR (cerebro*) OR (cerv*) OR (chem*) OR (chole*) OR (chro*) OR (cirr*) OR (clef*) OR (cont*) OR (corn*) OR (coro*) OR (crani*) OR (cryst*) OR (cyst*) OR (electro*) OR (emph*) OR (endo*) OR (exci*) OR (eye) OR (femo*) OR (fist*) OR (foot*) OR (gall*) OR (Gastric) OR (glau*) OR (glom*) OR (head*) OR (hear*) OR (heart*) OR (hip) OR (hipp*) OR (hyper*) OR (hypo*) OR (hyst*) OR (infection*) OR (inte*) OR (intra*) OR (join*) OR (kidn*) OR (lase*) OR (lipo*) OR (live*) OR (lump*) OR (lung*) OR (lymp*) OR (macro*) OR (marg*) OR (mech*) OR (meta*) OR (metas*) OR (micro*) OR (mitral*) OR (muta*) OR (neck*) OR (neph*) OR (nerv*) OR (neur*) OR (nutr*) OR (obes*) OR (onco*) OR (opht*) OR (orth*) OR (pancr*) OR (para*) OR (path*) OR (peni*) OR (peri*) OR (phaco*) OR (phar*) OR (pitu*) OR (plast*) OR (plat*) OR (post*) OR (prost*) OR (pulm*) OR (pump*) OR (radio*) OR (recon*) OR (rect*) OR (renal*) OR (reti*) OR (rib) OR (robo*) OR (sarcoma) OR (schw*) OR (spine*) OR (ster*) OR (stone) OR (stroke*) OR (testi*) OR (thor*) OR (throm*) OR (thyro*) OR (tibi*) OR (Ulce*) OR (uret*) OR (urol*) OR (vasc*) OR (venti*) OR (Tumor) OR (Suicide) OR (Vaccin*) OR (Malaria) OR (Mental NEXT health) OR (clinical NEXT trial*) OR (telemedicine) OR (abortion) OR (reproductive) OR (stillbirth) | 15,18,697 |
| 4 | #1 AND #2 NOT #3 | 446 |
|  | Limits: Jan 2014 to March 2024 | 306 |
|  | English | 293 |

**Cinahl**

| No. | Query | Output |
| --- | --- | --- |
| S1 | TI ((Surgical) OR (procedure*) OR (surger*) OR (operation*)) | 2,13,411 |
| S2 | TI ((volume) OR (rate*) OR (Enumerat*) OR (measur*) OR (modelling) OR (metrics) OR (unmet need*) OR (total number*)) | 2,19,593 |
| S3 | TI ((cardiac*) OR (adrenal) OR (aest*) OR (aneu*) OR (angio*) OR (ankl*) OR (anti*) OR (Aortic) OR (aqua*) OR (arte*) OR (arth*) OR (arthro*) OR (atria*) OR (augm*) OR (bari*) OR (bloo*) OR (brac*) OR (breast*) OR (calc*) OR (cancer*) OR (carci*) OR (cardio*) OR (cata*) OR (cerebro*) OR (cerv*) OR (chem*) OR (chole*) OR (chro*) OR (cirr*) OR (clef*) OR (cont*) OR (corn*) OR (coro*) OR (crani*) OR (cryst*) OR (cyst*) OR (electro*) OR (emph*) OR (endo*) OR (exci*) OR (eye) OR (femo*) OR (fist*) OR (foot*) OR (gall*) OR (Gastric) OR (glau*) OR (glom*) OR (head*) OR (hear*) OR (heart*) OR (hip) OR (hipp*) OR (hyper*) OR (hypo*) OR (hyst*) OR (infection*) OR (inte*) OR (intra*) OR (join*) OR (kidn*) OR (lase*) OR (lipo*) OR (live*) OR (lump*) OR (lung*) OR (lymp*) OR (macro*) OR (marg*) OR (mech*) OR (meta*) OR (metas*) OR (micro*) OR (mitral*) OR (muta*) OR (neck*) OR (neph*) OR (nerv*) OR (neur*) OR (nutr*) OR (obes*) OR (onco*) OR (opht*) OR (orth*) OR (pancr*) OR (para*) OR (path*) OR (peni*) OR (peri*) OR (phaco*) OR (phar*) OR (pitu*) OR (plast*) OR (plat*) OR (post*) OR (prost*) OR (pulm*) OR (pump*) OR (radio*) OR (recon*) OR (rect*) OR (renal*) OR (reti*) OR (rib) OR (robo*) OR (sarcoma) OR (schw*) OR (spine*) OR (ster*) OR (stone) OR (stroke*) OR (testi*) OR (thor*) OR (throm*) OR (thyro*) OR (tibi*) OR (Ulce*) OR (uret*) OR (urol*) OR (vasc*) OR (venti*) OR (Tumor) OR (Suicide) OR (Vaccin*) OR (Malaria) OR (Mental health) OR (clinical trial*) OR (telemedicine) OR (abortion) OR (reproductive) OR (stillbirth)) | 41,47,031 |
| S4 | S1 AND S2 | 7,157 |
| S5 | S4 NOT S3 | 1,882 |
| S6 | Publication date: Jan 2014-now, English language | 1,198 |
